# The 600-mm precipitation isoline distinguishes tree-ring-width responses to climate in China

**DOI:** 10.1093/nsr/nwy101

**Published:** 2018-09-14

**Authors:** Yu Liu, Huiming Song, Changfeng Sun, Yi Song, Qiufang Cai, Ruoshi Liu, Ying Lei, Qiang Li

**Affiliations:** 1 The State Key Laboratory of Loess and Quaternary Geology, Center for Excellence in Quaternary Science and Global Change, The Institute of Earth Environment, Chinese Academy of Sciences, Xi’an 710061, China; 2 Qingdao National Laboratory for Marine Science and Technology, Qingdao 266237, China; 3 Interdisciplinary Research Center of Earth Science Frontier and Joint Center for Global Change Studies, Beijing Normal University, Beijing 100875, China; 4 School of Human Settlements and Civil Engineering, Xi’an Jiaotong University, Xi’an 710049, China

**Keywords:** tree-ring width, climate response, 600-mm annual precipitation isoline, temperature, China

## Abstract

The numerous temperature and precipitation reconstructions in China based on tree-ring-width data have played significant roles in furthering the understanding of past climate changes. However, the geographical variability in the responses of trees to climate variations in China remains largely undetermined. Here, we describe an important spatial boundary in the response of trees to climate variations, namely the 600-mm annual precipitation isoline. We found that, to the north of this line, tree-ring widths are usually positively correlated with precipitation and negatively correlated with growing-season temperature. To the south of this line, the tree-ring widths respond positively to temperature, and winter half-year temperatures are the main reconstructed parameters, especially on the third topographical step of China. We also found that precipitation reconstructions based on tree-ring data and the Palmer Drought Severity Index almost exclusively fall in the region of the 200- to 600-mm annual precipitation isolines, not other regions. Our findings indicate that, when using multiple tree-ring-width chronologies for large-scale past climate reconstructions, the climatic signal of each tree-ring-width series should be carefully considered.

## INTRODUCTION

Tree rings have become an important source of data on past climatic and environmental change due to their high temporal resolution (annual or seasonal), accurate dating, wide geographical distribution and high continuity [1–6]. They play an important role in quantifying high-resolution climate changes during the past millennium. For example, temperature variations reconstructed primarily by tree-ring data from the Northern Hemisphere show the existence of the Little Ice Age, the Medieval Warm Period and global warming in the late twentieth century [4–6].

It is well known that moisture is an important factor that influences tree growth. Therefore, the amount of precipitation at a sampling site partly affects how trees respond to different climatic factors. China is mainly affected by the Asian monsoon [7,8], which is composed of a summer monsoon (warm and wet) phase and a winter monsoon (cold and dry) phase [8]. An important indicator of the strength of the summer monsoon is precipitation. Precipitation during the summer monsoon decreases along a gradient from the southeast to the northwest, which shapes the natural landscapes of the humid, sub-humid, semi-arid and arid regions from southeastern to northwestern China. The Xinjiang region of northwest China, where the Asian summer monsoon cannot penetrate, has a climate dominated by the westerlies and the climate of this region has distinct differences compared to other regions of China [9]. This paper focuses on the Asian summer monsoon climate zone that lies east of 90°E longitude.

**Figure 1.**
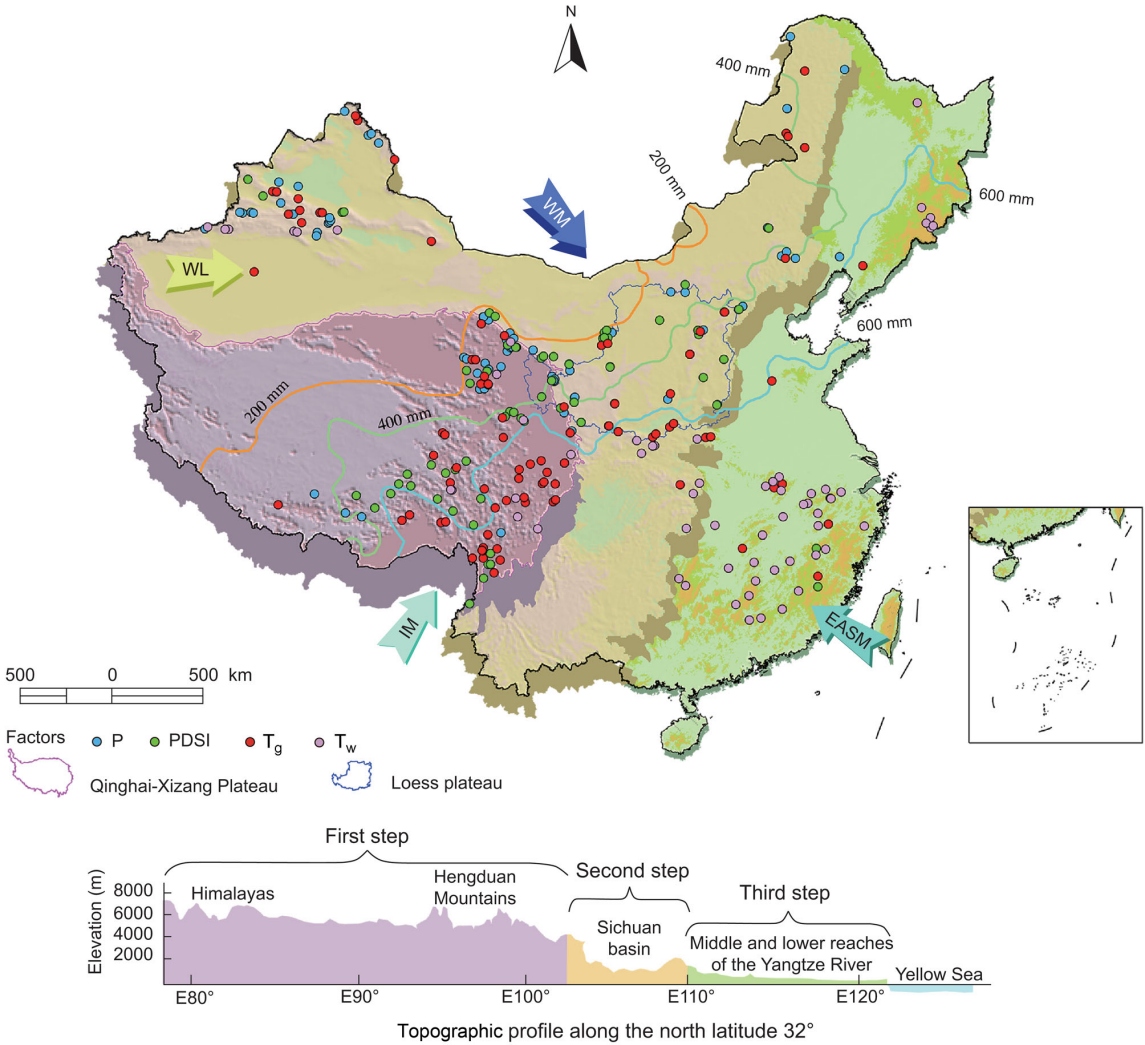
The geographical distribution of climatic factors reconstructed using tree-ring-width data across China. The purple region is the first geomorphic step, the yellow region is the second step and the green region is the third step. The blue dots denote precipitation (P) reconstruction; the green dots denote PDSI; the red dots represent the growing-season temperatures (T_g_); the pink dots are the winter half-year temperatures (T_w_). The orange line indicates the 200-mm/yr precipitation isoline; the green line denotes the 400-mm/yr; the blue line is the 600-mm/yr. Topographic profile along the 32°N latitude. All study sites are shown in Supplementary material, available as Supplementary Data at *NSR* online. This map was created using the software Arcview Version 3.3.

In China, many sequences of temperature, precipitation, Palmer Drought Severity Index (PDSI) and other variables have been reconstructed using single-site tree-ring-width data [10–15], which have established the foundation for large-scale climate reconstructions. Although the available data have not yet been fully utilized for large-scale climate integrations in China or Asia, a small subset of tree-ring data from China has been used in Asian climate reconstructions [1,2,16]. However, there are three topographical steps from east to west [17] and monsoon-related precipitation gradients decline from the southeast to the northwest in China [18] (Fig. 1). Therefore, the limitations of the tree-ring climate responses and the regional uncertainty of such reconstructions need to be investigated.

Here, we use data from 308 published tree-ring research sites in China (Fig. 1 and Supplementary Table 1, available as Supplementary Data at *NSR* online) to determine the tree–climate-response patterns. This paper uses climate responses presented in existing publications, rather than (re-)calculating climate responses for tree growth at any of the sites. After projecting the climate-response ‘type’ derived from tree-ring widths (TRWs) at each site on the map, we found that the 600-mm annual precipitation isoline (API), which divides the climate response into two geographical divisions, is a crucial boundary of tree-ring climate response. This study identified very significant spatial variability of the climatic response of TRWs in China. Therefore, we recommend that these differences should be considered when using TRW data for climate reconstruction, and suitable chronologies should be carefully selected to reconstruct different climate factors.

## DATA AND METHODS

### Data source

We collected published Chinese TRW-related articles before the end of 2016 from Elsevier, Springer, Wiley and CNKI (in Chinese). The articles were selected according to the following criteria: (i) studies were published in reputable peer-reviewed journals and (ii) studies produced definite relationships between TRWs and seasonal climatic data. Only those results with a correlation coefficient between ring-width index and climate variable of *r* > 0.54 with *P* < 0.05 were sufficiently strong to be part of our study—that is, the explained variance was larger than 29%. In total, 308 sites from 185 papers were selected for further review.

### Methods

The reconstructed climatic parameter and the correlation coefficients between tree-ring chronologies and climate obtained from all collected articles are shown in Supplementary Table 1, available as Supplementary Data at *NSR* online. Sites were marked on the map of China according to the climate response in order to identify patterns (Fig. 1). Based on the knowledge of Chinese climatic and topography (the three ‘steps’ in decline in elevation from west to east in China), after we projected all 308 available tree-ring sites and different annual precipitation isolines (e.g. 200, 300, 400, 500, 600, 800 and 1000 mm, etc.) on the map, we found that the 600-mm API is particularly important, which distinguishes the different tree-growth and climate-response patterns to the north and south.

## EASTERN SUB-REGION WITH AN API BETWEEN 200 AND 600 mm

This sub-region is part of the gradient of precipitation from semi-arid to arid, which corresponds to gradients of agriculture to animal husbandry and loess to desert areas [19] (on the second step). Monsoon precipitation is the main source of rainfall in this region. High temperature is often accompanied by lower precipitation and, conversely, more precipitation also corresponds to lower temperatures.

**Figure 2.**
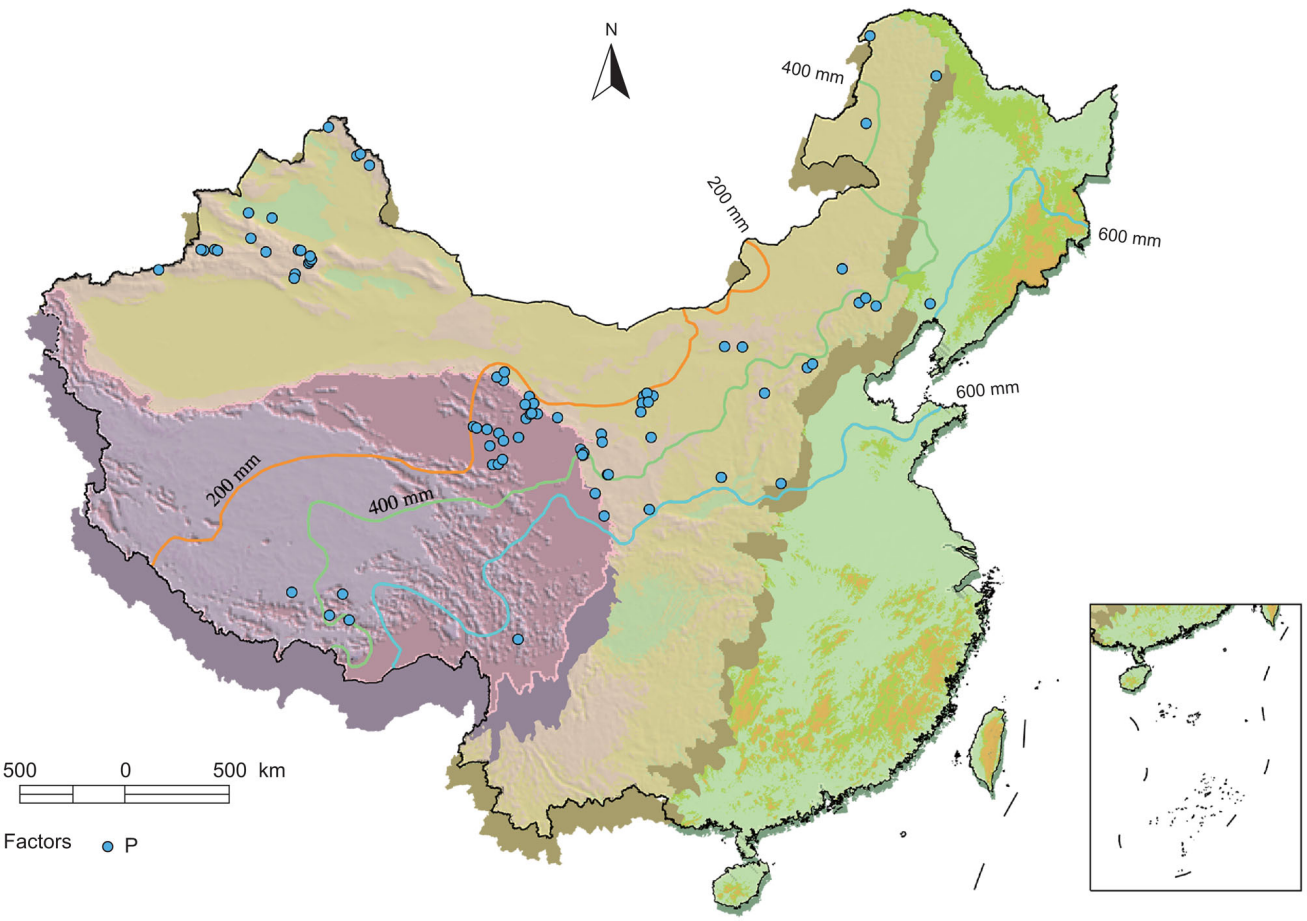
The distribution of the response relationship between tree-ring width and precipitation (P) (blue dots). The orange line indicates the 200-mm/yr precipitation isoline; the green line denotes the 400-mm/yr; the blue line is the 600-mm/yr. This map was created using the software Arcview Version 3.3.

Numerous dendrochronological studies have revealed a common positive response of TRWs to precipitation and a negative response to growing-season temperature in this region. Ninety (about 99% of total 91 sites) TRW-based precipitation reconstructions appear in the region between 200- and 600-mm API (Fig. 2), except for one site in this region exceeding 600-mm API. Thus, it is more reasonable to do large-scale precipitation integration in this precipitation-sensitive region than in the other more humid regions.

**Figure 3.**
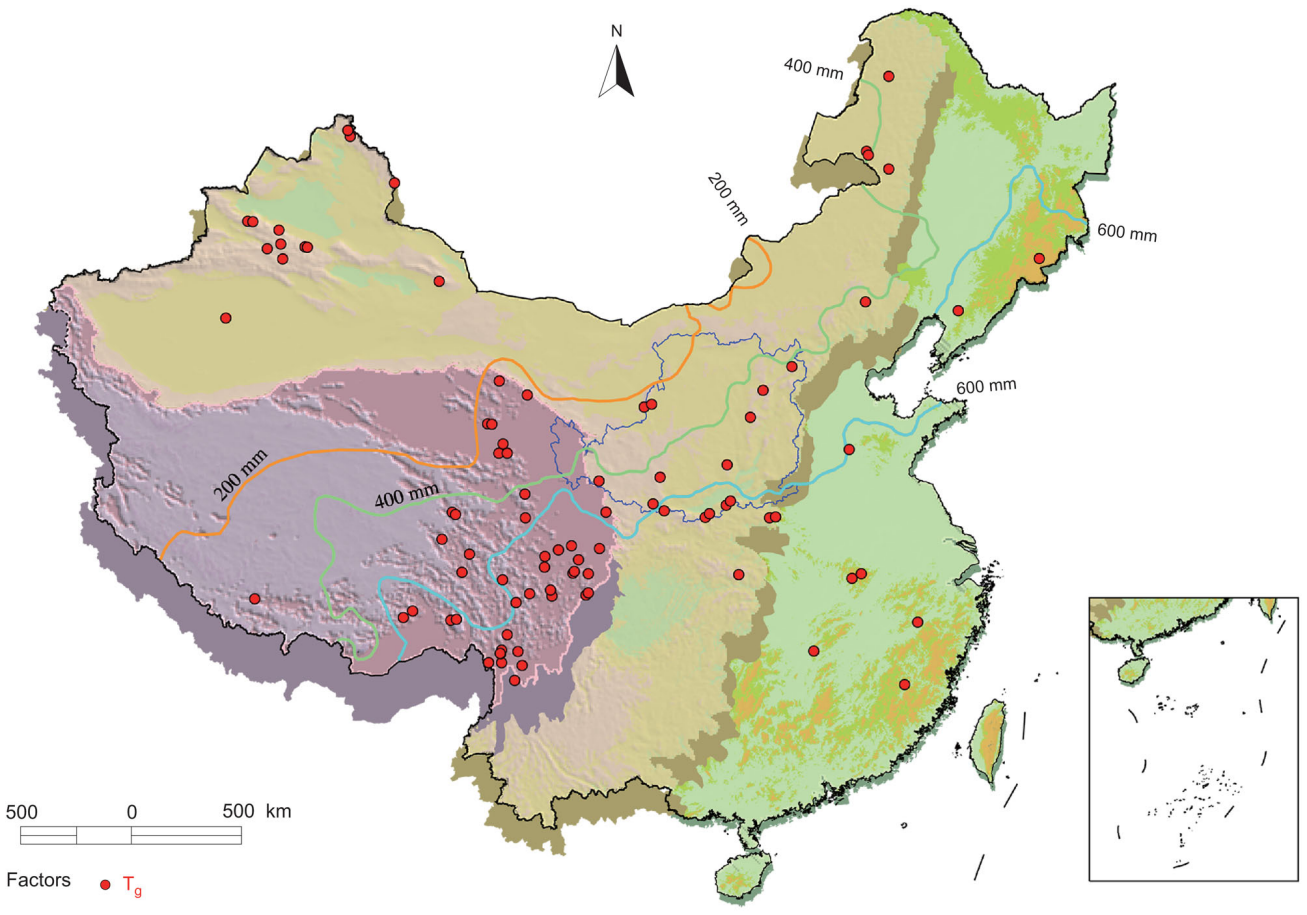
The distribution of the response relationship between tree-ring width and growing-season temperature (Tg). The orange line indicates the 200-mm/yr precipitation isoline; the green line denotes the 400-mm/yr; the blue line is the 600-mm/yr. This map was created using the software Arcview Version 3.3.

The pattern of TRWs positively correlated with precipitation and negatively correlated with temperature is typical in the semi-arid areas of China [20–24] (Figs 2 and 3). During the pre- and early growth seasons, there is less precipitation, since the monsoon has not yet fully arrived in this region [8]. High temperature accelerates not only the evaporation of soil moisture, which dries out soils, but also the transpiration rates in trees, which further depletes soil moisture and causes soil drought [25]. Severe drought contributes to water stress that limits or even stops radial growth [26]. However, there are few differences among the tree–climate-response patterns in this region.

In the region where annual precipitation is approximately 200–400 mm, the TRW has a stronger response to growing-season or annual precipitation than to temperature (Figs 2 and 3). For example, in Baotou [27], Baiyinaobao [28], Helan [29], Chifeng-Weichang [30], Hailaer [31], the Loess Plateau [32–34] and other western areas, the precipitation reconstructions based on tree-ring-width data show similar decadal variations [30,32], which jointly reflect the variations in the inland extent of the Asian summer monsoon in northern and northwestern China.

However, the limiting period of precipitation to tree growth shortens from the northwest to the southeast in the sub-region. For example, in the Changling–Shoulu, Liancheng and Xinglong Mountains in Gansu Province [32–34], TRW responds to the total precipitation throughout the year (from previous July to current June or from previous August to current July). In Baotou, TRW mainly responds to the total precipitation from February to July [27], while, in Xiaowutai, it responds to precipitation from February to May [35]. The well-known late-1920s extreme drought event in northern China was captured in most of the tree-ring records in these areas [30,32], indicating that this drought event was probably caused by a weakened Asian summer monsoon.

The trees living in areas with APIs of approximately 400–600 mm show extremely strong negative responses to the average temperature of the growing season, such as those in Kongtong [23], Shimen [36], Lvliang [37], Huanglong [38], Heng [39], Nanwutai [40] and Funiu [41]. In terms of tree physiology, the primary growing season for trees is from the late spring to early summer. If there is insufficient precipitation during this time interval, high temperatures will accelerate transpiration and lead to water stress that can limit net photosynthesis. High temperatures often reduce net photosynthesis by increasing the rate of respiration over the rate of photosynthesis, thereby reducing the accumulation of stored photosynthetic products, which results in the formation of narrow rings [26,42].

Tree-ring temperature reconstructions in this sub-region indicate relatively consistent variations at the decadal scale. The temperature reconstructions mentioned above not only show the same warm/cold intervals, but also reflect the characteristics of rapid heating and slow cooling, and jointly reveal late twentieth-century warming. The high temperatures in the 1920s to 1930s echoed the drought events during the same period in northern China. High temperatures generally accompanied droughts on the Chinese Loess Plateau, indicating that high temperatures enhance the effects of drought.

**Figure 4.**
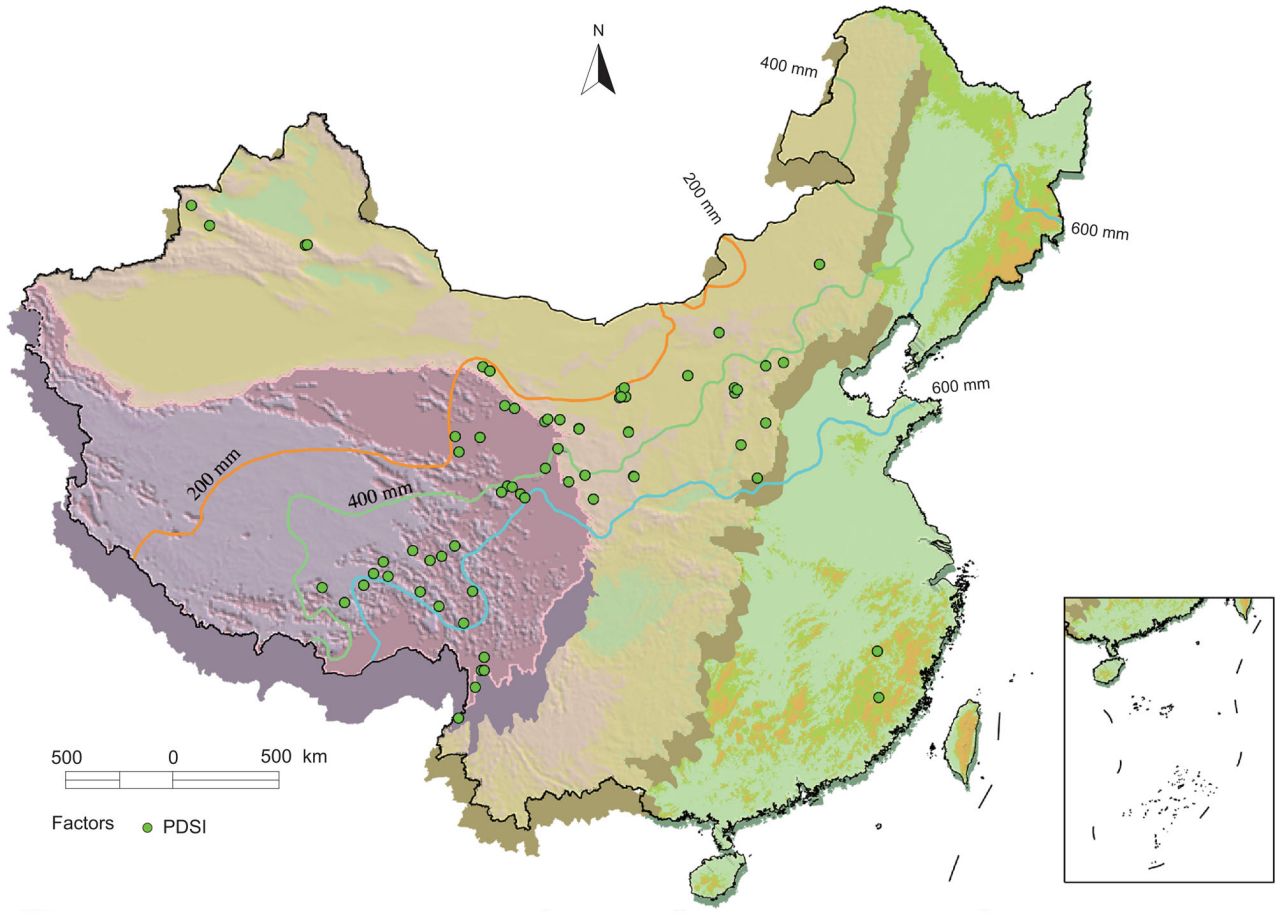
The distribution of the response relationship between tree-ring width and PDSI. The orange line indicates the 200-mm/yr precipitation isoline; the blue line is the 600-mm/yr. This map was created using the software Arcview Version 3.3.

In general, in the areas with an API within the range of 200–600 mm, TRW has a negative response to temperature and a positive response to precipitation, which makes it reasonable for tree rings to positively respond to the PDSI changes. TRW-PDSI responses almost entirely appear between the 200- and 600-mm API (Fig. 4). TRWs from some sites in the region were used to reconstruct past PDSI changes that reflect the drought history in northern China [22,23,43] and east of the Tibetan Plateau [44]. PDSI reflects the variations between dry–wet conditions and the changes in the strength of the Asian summer monsoon [45,46]. Undoubtedly, the region with an API between 200 and 600 mm is an appropriate choice to reconstruct a wide range of PDSI changes in China.

The factors that affect the climate of China are complex [47,48]; different climate systems simultaneously affect tree growth. Our analysis shows that precipitation reconstruction based on tree-ring data in the region with an API between 200 and 600 mm is not only influenced by the Asian summer monsoon [45], which generally contains a 2.5- to 2.8-year quasi-biennial oscillation (QBO characteristic) according to the characteristics of the Asian summer monsoon variations [49], but also influenced by other factors such as the Pacific Decadal Oscillation, El Niño-Southern Oscillation (ENSO) and solar activity. Since these sequences generally contain 3- to 5-year, 7- to 8-year, 11-year and centennial periodicities [12,50], synthetic influences by both ENSO and solar activity are also likely [45]. In addition, large-scale land–sea coupling is also reflected in the TRW records in this region, as they show very high correlation with the sea-surface temperatures of the tropical Indian Ocean, the equatorial Pacific Ocean, the East China Sea and the Sea of Japan [43], indicating that the sea-surface temperature changes surrounding China have a direct impact on the strength of the Asian summer monsoon, thereby affecting the changes in precipitation in the area. In addition to the above factors, the Pacific Decadal Oscillation [51] exhibits a degree of impact on the regional temperature changes, but the warming after 1850 is apparently associated with increasing human activities [52].

## WESTERN SUB-REGION WITH AN API BETWEEN 200 AND 600 mm

In the western sub-region with an API of 200–600 mm on the east Tibetan Plateau (on the first elevational step), TRWs are significantly positively correlated with precipitation throughout the year (from previous July to current June) [11,12] (Fig. 2) and show a significant, positive correlation with temperature (the whole year/winter half-year temperature) [10,53,54]. Due to the dry and cold climate on the plateau, the tree-ring chronologies here are more than 3000 years and the longest in China. Reconstructed precipitation series in this region are highly consistent with the temperature variations of the Northern Hemisphere on multi-decadal and longer time scales [11,13], and all of them show synchronous Medieval Warm Period [10,54,55], Little Ice Age [10,54–56] and global warming in the late twentieth century [10,54–56]. This indicates that the climate changes in the Northern Hemisphere are similar and driven by the same mechanism on a centennial scale.

## REGION WITH AN API GREATER THAN 600 mm

**Figure 5.**
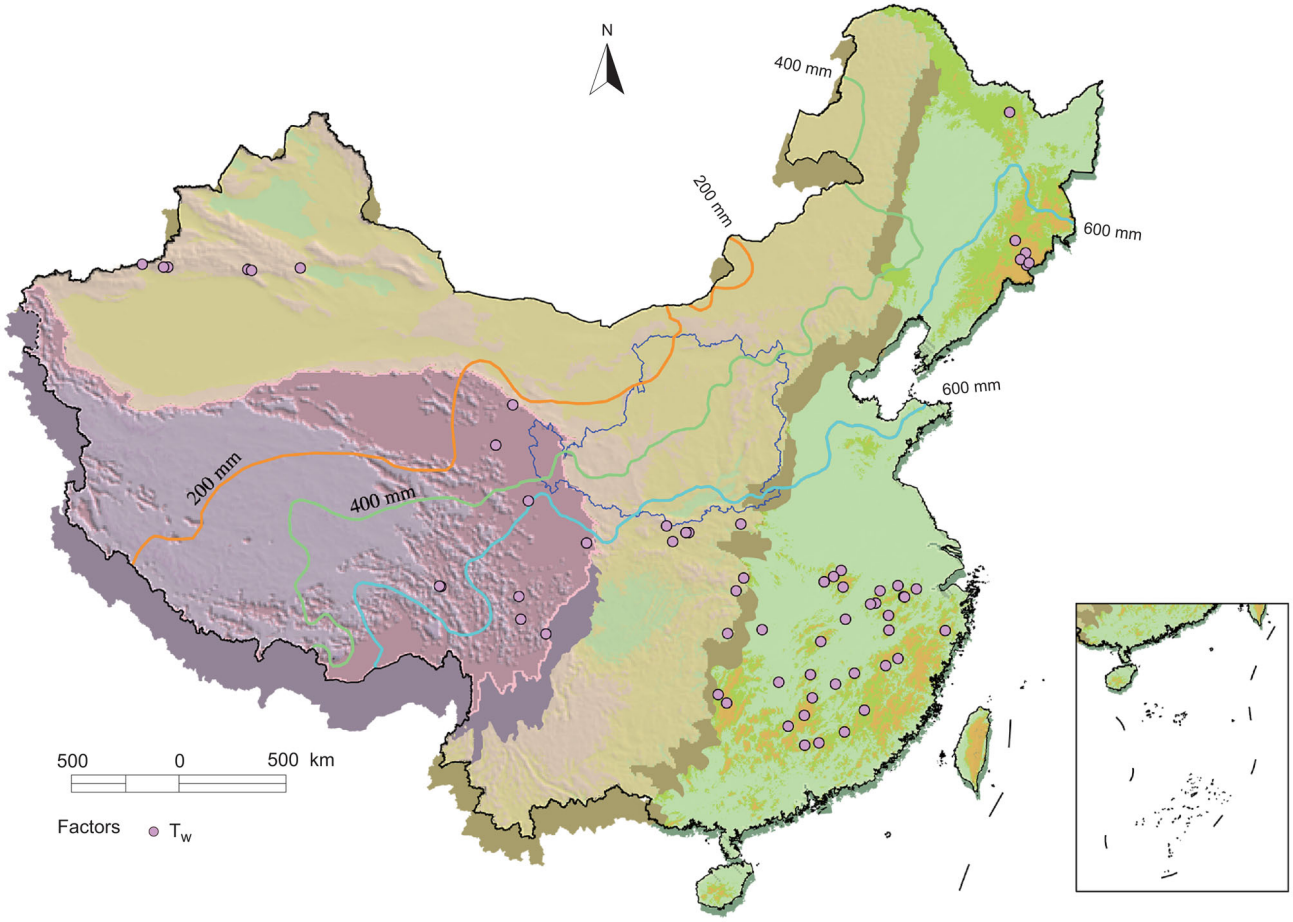
The distribution of the response relationship between tree-ring width and the temperature of winter half-year (T_w_). The orange line indicates the 200-mm/yr precipitation isoline; the green line denotes the 400-mm/yr; the blue line is the 600-mm/yr. This map was created using the software Arcview Version 3.3.

In the region where the API is greater than 600 mm, very few TRW data responded to precipitation (Fig. 2) or PDSI (Fig. 4), but most TRW data responded to temperature changes [57,58] (Figs 3 and 5).

In the first topographical step at a high altitude, the TRW data exhibit a typical positive response to the temperature during the growing season [59,60] or over the entire year [61–63] (Fig. 3). The low-frequency temperature changes are consistent with the glacier fluctuation cycles in the surrounding area [57,59,64]. Existing research results show that the trends of growing-season temperature, average annual temperature or other single seasonal temperatures are not consistent, and many of these trends do not show a regional warming trend [59,63]. Thus, whether these sequences are very suitable for temperature integration in China (or Asia or even globally) needs further investigation. We found that, for this region, only a few tree-ring chronologies responded to precipitation (Fig. 2) and PDSI changes (Fig. 4), namely the five sites located in the Hengduan Mountains and two other sites in southeastern China. The mean annual precipitation is slightly higher than 600 mm in southwest China. Drought usually happens in spring when the monsoon has not arrived. At the same time, more water is needed to support cambial activity. In this case, some trees respond significantly to PDSI variations.

In the vast region from southeast to northeast China on the third topographical step with an API greater than 600 mm, the TRW data mainly positively respond to the winter half-year temperature [65–70] (Fig. 5) and very few data series are negatively correlated with the growing-season temperatures [71–74]. This region is affected by the Asian summer monsoon and has the largest amount of annual precipitation in China, which easily meets the growth requirements of trees and is unlikely to be a limiting factor for growth. The relationship between climate and TRW is complicated, and false rings are very common; thus, advances in tree-ring climate research are more challenging in the region with rainfall larger than 600 mm. The limited amounts of currently published tree-ring data show that TRW exhibits a relatively similar response to climate changes in this area. While the TRWs respond to PDSI at only two sites [75], the remaining sites all respond to temperature (Figs 3 and 5). This might be caused by different tree species. One recent publication found that the Chinese subtropical pine ecosystem was more strongly regulated by net photosynthetic energy than environmental (climate) factors [76]. Whether this finding may be applicable to subtropical broadleaf species remains unsolved and merits further investigation.

In the third topographical step, the temperature data in the winter half-year weakly reflect the warming in the twentieth century [66,67], but some data do not reflect this warming [69]. This situation must be taken into consideration when building a regional model of temperature changes. Studies have shown that the reconstructed winter temperature in this area is mainly affected by the Asian winter monsoon [69], the atmospheric circulation at 500-hPa terrain height and the Siberian High [67].

This result clearly shows that, in the vast area of China where precipitation is greater than 600 mm/yr, the TRW data may be applied to reconstruct only regional temperature, but not precipitation or PDSI reconstructions. One more problem of tree-ring research in this area is that the tree-ring chronology is too short, at merely approximately 200 years, and thus is unfavorable for synthesizing long-term temperature data.

## CONCLUSION

To summarize, based on a now large number of studies, we propose an important boundary in monsoonal China to explain the climatic response differences of tree-ring width: namely the 600-mm API isoline. In the region north of the 600-mm API, precipitation is the main limiting factor of tree growth and TRWs have a positive response to the growth season or annual precipitation. In the eastern sub-region with an API between 200 and 600 mm, TRWs show extremely strong negative responses to temperature, while TRWs have a positive response to temperature in the western sub-region. South of the 600-mm API and on the first topographical step, temperature significantly affects tree growth and TRW shows a positive response to the growing-season temperature. In the region with precipitation greater than 600 mm on the third topographical step, TRW shows a positive correlation with winter half-year temperature.

It is worth noting that, in China, the climate systems are complicated, and the responses of TRWs to climate have distinct geographical distribution differences. Thus, when using multiple TRW chronologies for large-scale climate reconstructions, the climatic significance of TRW sequences across multiple regions should be fully considered. TRW data from a single site cannot be used to reliably reconstruct all climatic factors. To reconstruct the regional temperature, the selected chronologies should all have temperature or PDSI signals; to reconstruct a wide range of precipitation changes in China (or even in Asia), it may be feasible to select the chronologies that strongly respond to precipitation in the areas with API values below 600 mm. The general climatic characteristics of monsoonal China, namely ‘southern flood northern drought’ [77], determine the reconstructed precipitation curves in the region with an API below 600 mm in northern China, cannot represent the precipitation patterns of southern China and cannot represent precipitation more widely in Asia. Although it is urgent to understand the regional climate variability and mechanisms in the context of global change, cautious attention should be paid to the results of this study when a paleoclimate reconstruction is being planned. The tree-ring data from one site are not almighty.

## Supplementary Material

Supplemental Files
