# Supplementary material for: The 600-mm precipitation isoline distinguishes tree-ring-width responses to climate in China
Source: Natl Sci Rev. 2018 Sep 14;6(2):359–68. doi: 10.1093/nsr/nwy101 (PMC8291455; doi:10.1093/nsr/nwy101)
Supplement: Supplemental Files [file nwy101_supplemental_file.docx]

Supplementary Information for

**Title: The 600-mm precipitation isoline distinguishes tree-ring width responses to climate in China**

Authors: Yu Liu^1*, 2, 3, 4^ , Huiming Song^1, 2^, Changfeng Sun^1^, Yi Song^1^, Qiufang Cai^1^, Ruoshi Liu^4^ , Ying Lei^1^, Qiang Li^1^

Affiliations:

^1^ The State Key Laboratory of Loess and Quaternary Geology, The Institute of Earth Environment, Chinese Academy of Sciences, Xi’an 710061, China

^2^ Qingdao National Laboratory for Marine Science and Technology, Qingdao, 266237, China.

^3^ Interdisciplinary Research Center of Earth Science Frontier (IRCESF) and Joint Center for Global Change Studies (JCGCS), Beijing Normal University, Beijing 100875, China.

^4^ School of Human Settlements and Civil Engineering, Xi’an Jiaotong University, Xi’an 710049, China

***Correspondence to:**

Yu Liu,

Tel: 86–29–62336206; Fax: 86–29–62336234

Cell phone: 86–13359291673

[liuyu@loess.llqg.ac.cn](mailto:liuyu@loess.llqg.ac.cn), [liuyu@ieecas.cn](mailto:liuyu@ieecas.cn)

**Supplementary Tables:**

**Supplementary Table 1.**Information about the 309 tree-ring sites in China.

| No. | Site | Longitude (°E) | Latitude (°N) | Altitude (m) | Tree species | Climatic factor | Period | Correlation coefficient | P value | Ref. |
| --- | --- | --- | --- | --- | --- | --- | --- | --- | --- | --- |
| 1 | Changting, Fujian | 116.42 | 25.82 | 450–480 | *Pinus massoniana* | maxT_11_2_ | 1850–2009 | 0.57 | p<0.05 | [1] |
| 2 | Mt. Changbai | 128.07 | 42.12 | 1270–1750 | *Larix olgensis* | maxT_1_4_ | 1655–1993 | 0.76 | p<0.05 | [2] |
| 3 | Mt. Changbai | 128.2 | 42.6 | 650 | *Larix olgensis* | maxT_1_4_ | 1655–1993 | 0.76 | p<0.05 | [2] |
| 4 | Mt. Qianshan | 123.12 | 41 | 500–600 | *Pinus tabulaeformis* | maxT_5_7_ | 1745–2012 | 0.65 | p<0.0001 | [3] |
| 5 | Mt. Funiu | 111.97 | 33.7 | 1500–1700 | *Pinus tabulaeformis* | maxT_5_7_ | 1874–2007 | 0.63 | p<0.0001 | [4] |
| 6 | Daofu, Sichuan | 101.17 | 30.92 | 3750 | *Picea likiangensis* | maxT_6_ | 1617–1994 | 0.54 | p<0.001 | [5] |
| 7 | Seda, Sichuan | 100.82 | 32.27 | 3800 | *Picea likiangensis* | maxT_7_ | 1506–2008 | 0.63 | p<0.01 | [6] |
| 8 | Yulin, Shandong | 116.28 | 36.13 | 54 | *Pinus bangeana* | minT_10_9_ | 1616–2007 | 0.73 | p<0.0001 | [7] |
| 9 | Jiuzhaigou | 103.87 | 33.07 | 2998 | *Abies chensiensis* | minT_11_3_ | 1750–2003 | 0.68 | p<0.0001 | [8] |
| 10 | Xinlong, Sichuan | 100.7 | 30.87 | 3700 | *Picea likiangensis* | minT_1_4_ | 1650–1994 | 0.78 | p<0.01 | [9] |
| 11 | Yajiang, Sichuan | 100.85 | 29.97 | 3600 | *Picea likiangensis* | minT_1_4_ | 1650–1994 | 0.78 | p<0.01 | [9] |
| 12 | Liuba, Sichuan | 102.03 | 29.42 | 3900 | *Picea likiangensis* | minT_1_4_ | 1650–1994 | 0.78 | p<0.01 | [9] |
| 13 | Dabie Mountain | 116.2 | 31.12 | 1640–1760 | *Pinus taiwanensis* | minT_1_7_ | 1834–2011 | 0.76 | p<0.01 | [10] |
| 14 | Yanglonggou, Sichuan | 101.47 | 32.55 | 3680 | *Picea likiangensis* | minT_6_7_ | 1787–2005 | 0.77 | p<0.0001 | [11] |
| 15 | Sandaoping, Sichuan | 102.3 | 31.7 | 4070 | *Picea likiangensis* | minT_6_7_ | 1787–2005 | 0.77 | p<0.0001 | [11] |
| 16 | Tanglonggongma, Sichuan | 102.47 | 32.17 | 3650 | *Picea likiangensis* | minT_6_7_ | 1787–2005 | 0.77 | p<0.001 | [11] |
| 17 | Yong'an Fujian | 117.58 | 26.43 | 420–450 | *Pinus massoniana* | minT_7_10_ | 1803–2008 | –0.65 | p<0.05 | [12] |
| 18 | Ranwu Lake | 96.5 | 29.45 | 3982–4116 | *Picea wilsonii* | minT_8_ | 1385–2002 | 0.67 | p<0.05 | [13] |
| 19 | Ranwu Lake | 96.75 | 29.5 | 4087–4329 | *Picea wilsonii* | minT_8_ | 1385–2002 | 0.67 | p<0.05 | [13] |
| 20 | Hengduan Mountains | 99.93 | 29.15 | 3530 | *Abies forrestii* | P_9_6_ | 1509–2006 | 0.74 | p<0.01 | [14] |
| 21 | Gaoligong Mountain | 98.42 | 25.59 | 2930–80 | *Abies georgei* | PDSI_11_1_ | 1795–2004 | 0.62 | p<0.01 | [15] |
| 22 | central–Hengduan Mountains | 99 | 28.04 | 3100–3200 | *Picea wilsonii* | PDSI_3_5_ | 1655–2005 | 0.65 | p<0.01 | [16] |
| 23 | central–Hengduan Mountains | 99.33 | 27.59 | 3150–3240 | *Picea wilsonii* | PDSI_3_5_ | 1655–2005 | 0.65 | p<0.01 | [16] |
| 24 | Hengduan Mountains | 99.3 | 27.31 | 3040–3060 | *Abies forrestii* | PDSI_5_4_ | 1440–2007 | 0.61 | p<0.01 | [17] |
| 25 | Hengduan Mountains | 99.45 | 27.59 | 3050 | *Abies forrestii* | PDSI_5_4_ | 1440–2007 | 0.61 | p<0.01 | [17] |
| 26 | central–Hengduan Mountains | 98.96 | 28.24 | 3500–3600 | *Picea wilsonii* | T_10_9_ | 1750–2003 | 0.66 | p<0.01 | [18] |
| 27 | central–Hengduan Mountains | 98.99 | 28.38 | 4100 | *Picea wilsonii* | T_10_9_ | 1750–2003 | 0.66 | p<0.01 | [18] |
| 28 | central–Hengduan Mountains | 99.99 | 27.82 | 3540 | *Picea wilsonii* | T_10_9_ | 1750–2003 | 0.66 | p<0.01 | [18] |
| 29 | Xianyu Mountain | 117.28 | 30 | 1000–1051 | *Pinus taiwanensis* | T_12_3_ | 1852–2006 | 0.56 | p<0.05 | [19] |
| 30 | Tianmu Mountain | 118.9 | 30.1 | 1000–1050 | *Pinus taiwanensis* | T_12_3_ | 1852–2006 | 0.56 | p<0.05 | [19] |
| 31 | Funiu Mountain | 111.78 | 33.67 | 2100–2180 | *Pinus armandi* | T_12_4_ | 1911–2005 | 0.71 | p<0.001 | [20] |
| 32 | Fuping, Qinling Mountains | 107.8 | 33.72 | 2838 | *Abies chensiensis* | T_1_4_ | 1789–1993 | 0.70 | p<0.01 | [21] |
| 33 | Nanyue, Hunan | 112.7 | 27.27 | 603 | *Pinus massoniana* | T_1_4_ | 1851–2008 | 0.70 | p<0.0001 | [22] |
| 34 | Yizhang, Hunan | 112.97 | 25.47 | 502 | *Pinus massoniana* | T_1_4_ | 1851–2008 | 0.70 | p<0.0001 | [22] |
| 35 | Jinggangshan, Jiangxi | 114.15 | 26.53 | 866 | *Pinus massoniana* | T_1_4_ | 1851–2008 | 0.70 | p<0.0001 | [22] |
| 36 | Wugonshan, Jiangxi | 114.17 | 27.45 | 1431 | *Pinus massoniana* | T_1_4_ | 1851–2008 | 0.70 | p<0.0001 | [22] |
| 37 | Yangjiaping, Jiangxi | 114.78 | 28.73 | 941 | *Pinus massoniana* | T_1_4_ | 1851–2008 | 0.70 | p<0.0001 | [22] |
| 38 | middle–Qinling Mountains | 108.77 | 33.85 | 2600–2750 | *Larix gmelini* | T_1_7_ | 1814–2003 | 0.64 | p<0.0001 | [23] |
| 39 | Mt. Changbai | 127.78 | 43.18 | 765 | *Pinus koraiensis* | T_2_4_ | 1750–2002 | 0.68 | p<0.01 | [24] |
| 40 | Mt. Changbai | 127.83 | 42.4 | 870–940 | *Pinus koraiensis* | T_2_4_ | 1750–2002 | 0.68 | p<0.01 | [24] |
| 41 | Mt. Changbai | 128.25 | 42.2 | 1188 | *Pinus koraiensis* | T_2_4_ | 1750–2002 | 0.68 | p<0.01 | [24] |
| 42 | Dabie Mountain | 115.77 | 31.1 | 1500 | *Pinus taiwanensis* | T_2_7_ | 1869–2008 | 0.71 | p<0.05 | [25] |
| 43 | Zhenan, Qinling Mountains | 108.08 | 33.08 | 2200–2500 | *Abies chensiensis* | T_3_4_ | 1742–1991 | 0.71 | p<0.0001 | [26] |
| 44 | Zhenan, Qinling Mountains | 108.75 | 33.42 | 2500 | *Abies chensiensis* | T_3_4_ | 1775–1992 | 0.71 | p<0.0001 | [26] |
| 45 | Huanglong Mountain | 109.97 | 35.92 | 1370 | *Pinus tabulaeformis* | T_4_9_ | 1826–2004 | 0.63 | p<0.001 | [27] |
| 46 | Hua Mountain | 110.07 | 34.47 | 2082 | *Pinus tabulaeformis* | T_5_6_ | 1558–2006 | 0.64 | p<0.0001 | [28] |
| 47 | Nanwutai, Qinling Mountains | 108.97 | 34 | 1500–1600 | *Pinus tabulaeformis* | T_5_7_ | 1760–2005 | 0.67 | p<0.0001 | [29] |
| 48 | Gaoligong Mountain | 98.43 | 27.85 | 3300 | *Larix speciosa* | T_5_8_ | 1585–2004 | 0.60 | p<0.01 | [30] |
| 49 | Miyaluo, Sichuan | 102.97 | 31.62 | 3750 | *Sabina squamata* | T_6_7_ | 1810–2009 | 0.66 | p<0.01 | [31] |
| 50 | Balang Mountain | 102.87 | 30.75 | 3450 | *Abies recurvata* | T_6_8_ | 1850–2008 | 0.54 | p<0.01 | [32] |
| 51 | Sygera Mountain | 94.67 | 29.67 | 4280–4370 | *Abies georgei* | T_6_8_ | 1765–2006 | 0.59 | p<0.001 | [33] |
| 52 | Yunlin, Yunnan | 98.92 | 28.25 | 3400 | *Abies georgei* | T_6_8_ | 1710–2005 | 0.58 | p<0.01 | [34] |
| 53 | Badi, Yunnan | 99.03 | 27.88 | 3050 | *Abie sgeorgei* | T_6_8_ | 1710–2005 | 0.58 | p<0.01 | [34] |
| 54 | Tacheng, Yunnan | 99.37 | 27.38 | 2750 | *Abies georgei* | T_6_8_ | 1710–2005 | 0.58 | p<0.01 | [34] |
| 55 | Northwestern Yunnan | 99.77 | 28.37 | 3440 | *Picea brachytyla* | T_6_8_ | 1475–2003 | 0.69 | p<0.01 | [35] |
| 56 | Ayila Mountain | 102.1 | 32.73 | 3600–3800 | *Picea likiangensis* | T_7_ | 1597–2005 | 0.64 | p<0.01 | [36] |
| 57 | Mt. Changbai | 128.1 | 42.4 | 738 | *Pinus koraiensis* | T_9_10_ | 1765–2005 | 0.62 | p<0.001 | [37] |
| 58 | Muwang, Qinling Mountains | 108.88 | 33.43 | 2100 | *Pinus tabulaeformis* | T_9_4_ | 1837–2006 | 0.76 | p<0.0001 | [29] |
| 59 | Baokang, Hubei | 111.42 | 31.5 | 926 | *Pinus massoniana* | T_1_3_ | 1791–2008 | –0.70 | p<0.0001 | [38] |
| 60 | Macheng, Hubei | 115.23 | 31.08 | 1129 | *Pinus taiwanensis* | T_1_3_ | 1828–2008 | –0.70 | p<0.0001 | [38] |
| 61 | Ningguo, Anhui | 118.67 | 30.58 | 830 | *Pinus massoniana* | T_1_3_ | 1857–2007 | –0.70 | p<0.0001 | [38] |
| 62 | Qimen, Anhui | 117.53 | 30.02 | 280 | *Pinus massoniana* | T_1_3_ | 1861–2007 | –0.70 | p<0.0001 | [38] |
| 63 | Qingyang, Anhui | 117.8 | 30.48 | 589 | *Pinus massoniana* | T_1_3_ | 1896–2006 | –0.70 | p<0.0001 | [38] |
| 64 | Jixi, Anhui | 118.85 | 30.13 | 1456 | *Pinus taiwanensis* | T_1_3_ | 1863–2007 | –0.70 | p<0.0001 | [38] |
| 65 | Yuexi, Anhui | 116.1 | 30.8 | 1077 | *Pinus taiwanensis* | T_1_3_ | 1830–2007 | –0.70 | p<0.0001 | [38] |
| 66 | Tiantangzhai, Anhui | 115.77 | 31.12 | 1320 | *Pinus taiwanensis* | T_1_3_ | 1821–2007 | –0.70 | p<0.0001 | [38] |
| 67 | Mazongling, Anhui | 116.08 | 31.47 | 1460 | *Pinus taiwanensis* | T_1_3_ | 1878–2007 | –0.70 | p<0.0001 | [38] |
| 68 | Guidong, Hunan | 113.72 | 25.85 | 750 | *Pinus massoniana* | T_1_3_ | 1864–2008 | –0.70 | p<0.0001 | [38] |
| 69 | Zhangjiajie, Hunan | 110.5 | 29.35 | 757 | *Pinus massoniana* | T_1_3_ | 1846–2008 | –0.70 | p<0.0001 | [38] |
| 70 | Suining, Hunan | 110.28 | 26.57 | 403 | *Pinus massoniana* | T_1_3_ | 1901–2008 | –0.70 | p<0.0001 | [38] |
| 71 | Dongkou, Hunan | 115.25 | 26.98 | 864 | *Pinus massoniana* | T_1_3_ | 1812–2008 | –0.70 | p<0.0001 | [38] |
| 72 | Huitong, Hunan | 109.92 | 26.92 | 525 | *Pinus massoniana* | T_1_3_ | 1865–2008 | –0.70 | p<0.0001 | [38] |
| 73 | Yihuang, Jiangxi | 116.15 | 27.33 | 124 | *Pinus massoniana* | T_1_3_ | 1786–2008 | –0.70 | p<0.0001 | [38] |
| 74 | Yushan, Jiangxi | 117.97 | 28.88 | 908 | *Pinus massoniana* | T_1_3_ | 1810–2008 | –0.70 | p<0.0001 | [38] |
| 75 | Anyuan, Jiangxi | 115.43 | 25.02 | 704 | *Pinus massoniana* | T_1_3_ | 1848–2008 | –0.70 | p<0.0001 | [38] |
| 76 | Lushan, Jiangxi | 116.03 | 29.52 | 152 | *Pinus massoniana* | T_1_3_ | 1842–2008 | –0.70 | p<0.0001 | [38] |
| 77 | Lin’an, Zhengjiang | 119.52 | 30.35 | 750 | *Pinus massoniana* | T_1_3_ | 1791–2006 | –0.70 | p<0.0001 | [38] |
| 78 | Yongjia, Zhengjiang | 120.55 | 28.53 | 786 | *Pinus massoniana* | T_1_3_ | 1879–2007 | –0.70 | p<0.0001 | [38] |
| 79 | Xinchang, Zhengjiang | 112.12 | 29.4 | 380 | *Pinus massoniana* | T_1_3_ | 1771–2007 | –0.70 | p<0.0001 | [38] |
| 80 | Kaihua, Zhengjiang | 118.03 | 29.47 | 720 | *Pinus massoniana* | T_1_3_ | 1827–2007 | –0.70 | p<0.0001 | [38] |
| 81 | Tongmu, Fujian | 117.67 | 27.73 | 737 | *Pinus massoniana* | T_1_3_ | 1809–2008 | –0.70 | p<0.0001 | [38] |
| 82 | Dehua, Fujian | 118.22 | 27.7 | 770 | *Pinus massoniana* | T_1_3_ | 1854–2008 | –0.70 | p<0.0001 | [38] |
| 83 | Qujiang, Guangdong | 113.63 | 24.65 | 128 | *Pinus massoniana* | T_1_3_ | 1941–2008 | –0.70 | p<0.0001 | [38] |
| 84 | Shixing, Guangdong | 114.25 | 24.7 | 515 | *Pinus massoniana* | T_1_3_ | 1905–2008 | –0.70 | p<0.0001 | [38] |
| 85 | Qumacai, Qinghai | 96.13 | 33.8 | 4060 | *Sabina tibetica* | maxT_4_6_ | 1550–2002 | 0.62 | p<0.05 | [39] |
| 86 | Yeduo, Qinghai | 96.28 | 33.72 | 3950 | *Sabina tibetica* | maxT_4_6_ | 1550–2002 | 0.62 | p<0.05 | [39] |
| 87 | Animaqin Mountains | 99.78 | 33.78 | 3540 | *Sabina przewalskii* | maxT_4_9_ | 1172–2001 | –0.68 | p<0.005 | [40] |
| 88 | Animaqin Mountains | 99.68 | 34.73 | 3600–3700 | *Sabina przewalskii* | maxT_4_9_ | 1334–2001 | 0.67 | p<0.01 | [41] |
| 89 | Zaduo, Qinghai | 95.72 | 32.67 | 4200–4230 | *Sabina tibetica* | maxT_5_6_ | 1360–2005 | -0.77 | p<0.001 | [42] |
| 90 | Changdu, Tibet | 96.97 | 31.07 | 4290 | *Picea likiangensis* | minT_10_1_ | 1594–2006 | 0.66 | p<0.001 | [43] |
| 91 | Xiqing Mountain | 100.77 | 34.77 | 3500–3615 | *Sabina przewalskii* | minT_10_4_ | 1577–2001 | 0.63 | p<0.01 | [44] |
| 92 | Yushu, Qinghai | 97.13 | 32.17 | 3650 | *Picea likiangensis* | minT_6_8_ | 1624–2002 | 0.52 | p<0.001 | [45] |
| 93 | Huanglong Mountain | 109.78 | 35.65 | 1427 | *Pinus tabulaeformis* | P_2_3_ | 1810–2002 | 0.63 | p<0.001 | [46] |
| 94 | Xiaowutai Mountain | 114.68 | 39.75 | 1553 | *Pinus tabulaeformis* | P_2_5_ | 1895–2005 | 0.69 | p<0.001 | [47] |
| 95 | Xiaowutai Mountain | 114.95 | 39.87 | 1600 | *Pinus tabulaeformis* | P_2_5_ | 1895–2005 | 0.69 | p<0.001 | [47] |
| 96 | Xiaolong Mountain | 106.13 | 34.45 | 2660 | *Pinus tabulaeformis* | P_4_7_ | 1607–2009 | 0.67 | p<0.01 | [48] |
| 97 | south central Tibet | 91.03 | 29.38 | 4275–4420 | *Sabina tibetica* | P_7_6_ | 1480–2007 | 0.59 | p<0.01 | [49 |
| 98 | south central Tibet | 91.97 | 29.3 | 4434–4550 | *Sabina tibetica* | P_7_6_ | 1480–2007 | 0.59 | p<0.01 | [49] |
| 99 | Linzhou, Tibet | 91.5 | 30.3 | 4200–4557 | *Juniperus rigida* | P_7_6_ | 1037–2009 | 0.71 | p<0.05 | [50] |
| 100 | Xinglong Mountain | 104.07 | 35.78 | 2314 | *Picea crassifolia* | P_7_6_ | 1679–2008 | 0.73 | p<0.0001 | [51] |
| 101 | Lazikou, Gansu | 103.9 | 34.2 | 2150–2450 | *Pinus tabulaeformis* | P_8_7_ | 1824–2011 | 0.64 | p<0.01 | [52] |
| 102 | Chifeng, Inner Mongolia | 118.05 | 42.07 | 1000–1100 | *Pinus tabulaeformis* | P_8_7_ | 1777–2003 | 0.69 | p<0.0001 | [53] |
| 103 | Weichang, Hebei | 118.17 | 42.12 | 1000 | *Pinus tabulaeformis* | P_8_7_ | 1777–2003 | 0.69 | p<0.0001 | [53] |
| 104 | Mt. Guancen | 112.08 | 38.83 | 1600–2787 | *Pinus tabulaeformis* | P_8_7_ | 1686–2003 | 0. 70 | p<0.0001 | [54] |
| 105 | Tibet Nakqu Region | 93.46 | 30.6 | 4233 | *Sabina tibetica* | PDSI_3_6_ | 1573–2004 | 0.66 | p<0.0001 | [55] |
| 106 | Tibet Nakqu Region | 94.29 | 31.63 | 3854 | *Sabina tibetica* | PDSI_3_6_ | 1573–2004 | 0.66 | p<0.0001 | [55] |
| 107 | central–Taihang Mountains | 113.45 | 37.4 | 1440–1500 | *Pinus tabulaeformis* | PDSI_5_6_ | 1873–2008 | 0.66 | p<0.0001 | [56] |
| 108 | Kongtong Mountain | 106.53 | 35.52 | 1500–2123 | *Pinus tabulaeformis* | PDSI_5_7_ | 1723–2005 | –0.82 | p<0.0001 | [57] |
| 109 | Kongtong Mountain | 106.51 | 35.54 | 1800 | *Pinus tabulaeformis* | PDSI_5_7_ | 1615–2009 | 0.84 | p<0.01 | [58] |
| 110 | Guiqing Mountain | 104.47 | 34.63 | 2436 | *Pinus tabulaeformis* | PDSI_5_8_ | 1618–2005 | 0.70 | p<0.01 | [59] |
| 111 | Xinglong Mountain | 104.03 | 35.67 | 2370–2570 | *Piceawilsonii* | PDSI_8_7_ | 1794–2003 | 0.63 | p<0.01 | [60] |
| 112 | Wuying, Heilongjiang | 129.2 | 48.23 | 360–460 | *Pinus koraiensis* | pT_10_ | 1796–2004 | 0.59 | p<0.0001 | [61] |
| 113 | Guancen Mountain | 111.97 | 38.7 | 1750–1874 | *Pinus tabulaeformis* | PDSI_4_7_ | 1853–2008 | 0.73 | p<0.0001 | [62] |
| 114 | Guancen Mountain | 112.07 | 38.85 | 16801780 | *Pinus tabulaeformis* | PDSI_4_7_ | 1853–2008 | 0.73 | p<0.0001 | [62] |
| 115 | Linzhi, Tibet | 94.27 | 29.37 | 3100–3400 | *Cupressus gigantea* | T_1_6_ | 1377–1998 | 0.59 | p<0.001 | [63] |
| 116 | Kongtong Mountain | 106.53 | 35.52 | 1500–2123 | *Pinus tabulaeformis* | T_2_9_ | 1723–2005 | –0.66 | p<0.0001 | [64] |
| 117 | Leiwuqi, Qinghai | 96.83 | 31.42 | 3996 | *Sabina tibetica* | T_5_6_ | 1440–2006 | –0.80 | p<0.001 | [65] |
| 118 | Heng Mountains | 113.73 | 39.67 | 1710–1780 | *Pinus tabulaeformis* | T_5_6_ | 1767–2008 | –0.71 | p<0.001 | [66] |
| 119 | Weichang, Hebei | 118.07 | 42.07 | 1256 | *Pinus tabulaeformis* | T_5_6_ | 1884–2002 | 0.66 | p<0.0001 | [67] |
| 120 | Beiwudangshan Mountain | 111.33 | 37.77 | 1740–1900 | *Pinus tabulaeformis* | T_5_7_ | 1836–2003 | –0.67 | p<0.001 | [68] |
| 121 | Ningwu, Shanxi | 112.08 | 38.83 | 1600–2100 | *Pinus tabulaeformis* | T_5_7_ | 1779–2003 | 0.67 | p<0.0001 | [69] |
| 122 | Kongtong Mountain | 106.53 | 35.52 | 1910–1980 | *Pinus tabulaeformis* | T_6_8_ | 1751–2005 | –0.62 | p<0.01 | [70] |
| 123 | Anren, Tibet | 87.18 | 29.25 | 4500–4750 | *Sabina tibetica* | T_7_6_ | 1612–1998 | 0.64 | p<0.001 | [71] |
| 124 | Yiwulv Mountain | 121.7 | 41.62 | 415 | *Pinus tabulaeformis* | P_9_7_ | 1818–2010 | 0.60 | p<0.0001 | [72] |
| 125 | Hulunbuir, Inner Mongolia | 119.38 | 48 | 760–790 | *Pinus sylvestris* | maxT_4_9_ | 1868–2008 | –0.64 | p<0.0001 | [73] |
| 126 | Hulunbuir, Inner Mongolia | 119.48 | 47.83 | 894–915 | *Pinus sylvestris* | maxT_4_9_ | 1868–2008 | –0.64 | p<0.0001 | [73] |
| 127 | Delingha, Qinghai | 97.55 | 37.45 | 3920 | *Sabina przewalskii* | maxT_5_6_ | 691–2002 | –0. 71 | p<0.01 | [74] |
| 128 | Delingha, Qinghai | 97.78 | 37.45 | 3700 | *Sabina przewalskii* | maxT_5_6_ | 757–2002 | –0. 71 | p<0.01 | [74] |
| 129 | Jiuquan, Gansu | 98.1 | 39.55 | 3000–3100 | *Picea crassifolia* | maxT_6_9_ | 1768–2007 | –0.68 | p<0.01 | [75] |
| 130 | south–central Helan Mountains | 105.77 | 38.52 | 2400–2500 | *Pinus tabulaeformis* | P_1_7_ | 1775–1998 | 0.73 | p<0.0001 | [76] |
| 131 | south–central Helan Mountains | 105.98 | 38.72 | 2500–2600 | *Pinus tabulaeformis* | P_1_7_ | 1775–1998 | 0.73 | p<0.0001 | [76] |
| 132 | Helan Mountains | 105.77 | 38.52 | 2400–2500 | *Pinus tabulaeformis* | P_2_7_ | 1802–1997 | 0.68 | p<0.001 | [77] |
| 133 | Helan Mountains | 105.98 | 38.72 | 2500–2600 | *Pinus tabulaeformis* | P_2_7_ | 1802–1997 | 0.68 | p<0.001 | [77] |
| 134 | Helan Mountains | 106.08 | 39.08 | 1500–2000 | *Pinus tabulaeformis* | P_2_7_ | 1802–1997 | 0.68 | p<0.001 | [77] |
| 135 | Wudangzhao, Neimemggu | 110.33 | 40.83 | 1500–1800 | *Pinus tabulaeformis* | P_2_7_ | 1734–2001 | 0.67 | p<0.0001 | [78] |
| 136 | Lamadong, Inner Mongolia | 111.28 | 40.77 | 1300–1600 | *Pinus tabulaeformis* | P_2_7_ | 1627–2001 | 0.65 | p<0.0001 | [78] |
| 137 | Liancheng, Gansu | 102.77 | 36.6 | 2900–3100 | *Sabina przewalskii* | P_3_4_ | 1719–1998 | 0.53 | p<0.01 | [79] |
| 138 | Shidalong, Gansu | 99.95 | 38.45 | 2700–2800 | *Picea crassifolia* | P_3_5_ | 1765–1995 | 0.59 | p<0.01 | [80] |
| 139 | Baiyinaobao, Inner Mongolia | 117.18 | 43.52 | 1400–1450 | *Picea koraiensis* | P_4_7_ | 1838–1999 | 0.70 | p<0.0001 | [81] |
| 140 | Nansi | 105.83 | 38.68 | 2240–2280 | *Pinus tabulaeformis* | P_5_7_ | 1775–2005 | 0.61 | p<0.001 | [82] |
| 141 | Beisi | 105.92 | 38.97 | 2205–2210 | *Pinus tabulaeformis* | P_5_7_ | 1775–2005 | 0.61 | p<0.001 | [82] |
| 142 | Daxigou | 105.95 | 38.98 | 2100–2300 | *Pinus tabulaeformis* | P_5_7_ | 1775–2005 | 0.61 | p<0.001 | [82] |
| 143 | Beisigou | 106.08 | 39.08 | 1500–2000 | *Pinus tabulaeformis* | P_5_7_ | 1726–1997 | 0.65 | p<0.0001 | [83] |
| 144 | Helan Mountain | 106.08 | 39.08 | 1500–2000 | *Pinus tabulaeformis* | P_5_7_ | 1740–1997 | 0 .68 | p<0.05 | [84] |
| 145 | Qilian Mountains | 98.45 | 39.42 | 3050–3090 | *Sabina przewalskii* | P_6_ | 1803–2006 | 0.67 | p<0.01 | [85] |
| 146 | Qilian Mountains | 98.48 | 39.77 | 2800–2900 | *Sabina przewalskii* | P_6_ | 1803–2006 | 0.67 | p<0.01 | [85] |
| 147 | Hexi Corridor | 98.08 | 39.55 | 3000–3500 | *Sabina przewalskii* | P_7_6_ | 1390–2007 | 0.59 | p<0.01 | [86] |
| 148 | eastern Qaidam Basin | 98.67 | 37.03 | 3740–3800 | *Sabina przewalskii* | P_7_6_ | 1219–2002 | 0.62 | p<0.001 | [87] |
| 149 | Changling Mountain | 103.7 | 37.45 | 2400–2600 | *Pinus tabulaeformis* | P_7_6_ | 1853–2007 | 0.64 | p<0.0001 | [88] |
| 150 | Mt. Shoulong | 103.73 | 37.13 | 2498 | *Pinus tabulaeformis* | P_7_6_ | 1853–2007 | 0.64 | p<0.0001 | [88] |
| 151 | Qilian ,Qinghai | 100.33 | 38.17 | 3140–3390 | *Picea crassifolia* | P_7_6_ | 1753–2000 | 0.65 | p<0.0001 | [89] |
| 152 | Qilian Mountains | 98.1 | 39.55 | 3000–3150 | *Picea crassifolia* | P_7_6_ | 1768–2009 | 0.67 | p<0.05 | [90] |
| 153 | Zhamashike, QinlingMontains | 100.03 | 38.18 | 3300 | *Sabina przewalskii* | P_7_6_ | 1634–2000 | 0.69 | p<0.0001 | [91] |
| 154 | Central–Qilian Mountains | 99.78 | 38.23 | 3300 | *Sabina przewalskii* | P_7_6_ | 1063–2009 | 0.70 | p<0.0001 | [92] |
| 155 | Central–Qilian Mountains | 99.95 | 38.15 | 3300 | *Sabina przewalskii* | P_7_6_ | 1063–2009 | 0.70 | p<0.0001 | [92] |
| 156 | Dulan, Qinghai | 98.18 | 36.05 | 3800–4200 | *Sabina przewalskii* | P_7_6_ | 850–2000 | 0.68 | p<0.0001 | [93] |
| 157 | Dulan, Qinghai | 98.47 | 36.08 | 3800–4000 | *Sabina przewalskii* | P_7_6_ | 850–2000 | 0.68 | p<0.0001 | [93] |
| 158 | Dulan, Qinghai | 98.67 | 36.28 | 4000–4200 | *Sabina przewalskii* | P_7_6_ | 850–2000 | 0.68 | p<0.0001 | [93] |
| 159 | Hailar, Inner Mongolia | 119.72 | 49.2 | 450–600 | *Pinus sylvestris* | P_7_6_ | 1865–2003 | 0.71 | p<0.0001 | [94] |
| 160 | Shalike Mountain | 97.23 | 37.47 | 3730–3780 | *Sabina przewalskii* | P_7_6_ | 1002–2001 | 0.68 | p<0.0001 | [95] |
| 161 | Shalike Mountain | 97.78 | 37.45 | 3700 | *Sabina przewalskii* | P_7_6_ | 1002–2001 | 0.68 | p<0.0001 | [95] |
| 162 | Zongwulong Mountain | 98.32 | 36.72 | 3700 | *Sabina przewalskii* | P_7_6_ | 1002–2001 | 0.68 | p<0.0001 | [95] |
| 163 | Qaidam Basin | 97.05 | 37.52 | 3780 | *Sabina przewalskii* | P_7_6_ | 566–2000 | 0.81 | p<0.01 | [96] |
| 164 | Qaidam Basin | 98.4 | 37.32 | 3500 | *Sabina przewalskii* | P_7_6_ | 566–2000 | 0.81 | p<0.01 | [96] |
| 165 | Qaidam Basin | 98.65 | 37.03 | 3700 | *Sabina przewalskii* | P_7_6_ | 566–2000 | 0.81 | p<0.01 | [96] |
| 166 | Shandan, Gansu | 101.4 | 38.07 | 3000–3100 | *Picea crassifolia* | P_8_6_ | 1783–2006 | 0.65 | p<0.0001 | [97] |
| 167 | Huzu | 102.65 | 36.83 | 2170–2270 | *Pinus tabulaeformis* | P_8_6_ | 1821–2009 | 0.71 | p<0.001 | [98] |
| 168 | Kongganglin | 99.73 | 38.8 | 2900–3150 | *Sabina przewalskii* | P_8_7_ | 1480–1999 | 0.61 | p<0.001 | [99] |
| 169 | Sunan | 99.7 | 38.68 | 2900–3200 | *Sabina przewalskii* | P_8_7_ | 775–2006 | 0.62 | p<0.001 | [100] |
| 170 | Liancheng, Gansu | 102.73 | 36.69 | 2146 | *Pinus tabulaeformis* | P_8_7_ | 1777–2008 | 0.67 | p<0.0001 | [101] |
| 171 | Liancheng, Gansu | 102.75 | 36.62 | 2370 | *Pinus tabulaeformis* | P_8_7_ | 1777–2008 | 0.67 | p<0.0001 | [101] |
| 172 | Kalaqin, Inner Mongolia | 118.78 | 41.87 | 1108–1198 | *Pinus tabulaeformis* | P_8_7_ | 1771–2008 | 0.67 | p<0.0001 | [102] |
| 173 | Hulunbuir, Inner Mongolia | 119.7 | 49.2 | 515–669 | *Pinus sylvestris* | P_8_7_ | 1829–2009 | 0.78 | p<0.0001 | [103] |
| 174 | Changling Mountain | 103.68 | 37.45 | 2500–2550 | *Pinus tabulaeformis* | P_9_7_ | 1691–2006 | 0.69 | p<0.001 | [104] |
| 175 | Luo Mountain | 106.27 | 37.32 | 2400 | *Pinus tabulaeformis* | P_9_8_ | 1899–2007 | 0.56 | 0.001 | [105] |
| 176 | Changling Mountain | 103.68 | 37.45 | 2500–2533 | *Pinus tabulaeformis* | P_9_8_ | 1860–2000 | 0.65 | p<0.0001 | [106] |
| 177 | NorternHelan Mountains | 105.98 | 38.72 | 1900–2400 | *Pinus tabulaeformis* | PDSI_3_7_ | 1788–1999 | 0.68 | p<0.0001 | [107] |
| 178 | NorternHelan Mountains | 106.08 | 39.08 | 2000–2350 | *Pinus tabulaeformis* | PDSI_3_7_ | 1788–1999 | 0.68 | p<0.0001 | [107] |
| 179 | Qilian, Mountain | 98.48 | 39.77 | 2000–3000 | *Sabina przewalskii* | PDSI_3_9_ | 1855–2001 | 0.65 | p<0.001 | [108] |
| 180 | Changling Mountain[ | 103.68 | 37.45 | 2500–2533 | *Pinus tabulaeformis* | PDSI_5_6_ | 1691–2005 | 0.65 | p<0.01 | [109] |
| 181 | Qilian Mount[ain | 102.72 | 36.68 | 2400–2500 | *Pinus tabulaeformis* | PDSI_5_7_ | 1862–2007 | 0.66 | p<0.001 | [110] |
| 182 | Qilian Mountain | 103.68 | 37.43 | 2400–2600 | *Pinus tabulaeformis* | PDSI_5_7_ | 1862–2007 | 0.66 | p<0.001 | [110] |
| 183 | Qilian Mountain | 106.27 | 37.3 | 2400 | *Pinus tabulaeformis* | PDSI_5_7_ | 1862–2007 | 0.66 | p<0.001 | [110] |
| 184 | Nansi | 105.83 | 38.68 | 2240–2280 | *Pinus tabulaeformis* | PDSI_5_7_ | 1759–2005 | 0.69 | p<0.001 | [111] |
| 185 | Suyukou | 105.92 | 38.73 | 2260–2320 | *Pinus tabulaeformis* | PDSI_5_7_ | 1759–2005 | 0.69 | p<0.001 | [111] |
| 186 | Beisi | 105.92 | 38.97 | 2205–2210 | *Pinus tabulaeformis* | PDSI_5_7_ | 1759–2005 | 0.69 | p<0.001 | [111] |
| 187 | Ortindag Sand Land | 117.25 | 43.5 | 1200–1360 | *Pinus tabulaeformis* | PDSI_5_7_ | 1842–2002 | 0.72 | p<0.001 | [112] |
| 188 | Luo Mountain | 106.27 | 37.3 | 2400 | *Pinus tabulaeformis* | PDSI_8_7_ | 1897–2004 | 0.56 | p<0.001 | [113] |
| 189 | baishu | 101.91 | 37.7 | 2740–70 | *Picea crassifolia* | PDSI_9_8_ | 1856–2009 | 0.71 | p<0.01 | [114] |
| 190 | Longtan | 102.05 | 37.8 | 2680–2720 | *Picea crassifolia* | PDSI_9_8_ | 1856–2009 | 0.71 | p<0.01 | [114] |
| 191 | Daan | 102.7 | 37.79 | 3072–3085 | *Picea crassifolia* | PDSI_9_8_ | 1856–2009 | 0.71 | p<0.01 | [114] |
| 192 | Wulan–Dulan | 98.67 | 36.33 | 3300 | *Sabina przewalskii* | pT_1_12_ | bc485–2000 | 0.69 | p<0.0001 | [115] |
| 193 | middle Qilian Mountains | 99.77 | 38.27 | 3300 | *Sabina przewalskii* | PDSI_5_7_ | 1560–2009 | 0.69 | p<0.0001 | [116] |
| 194 | middle Qilian Mountains | 100.27 | 38.18 | 3200–3300 | *Sabina przewalskii* | PDSI_5_7_ | 1560–2009 | 0.69 | p<0.0001 | [116] |
| 195 | Sunan | 99.93 | 38.43 | 3400–3550 | *Sabina przewalskii* | T_12_4_ | 1000–2000 | 0.58 | p<0.001 | [117] |
| 196 | Yaoba | 105.77 | 38.52 | 2400–2500 | *Pinus tabulaeformis* | T_1_8_ | 1776–1999 | 0.66 | p<0.001 | [118] |
| 197 | Yaoba | 105.98 | 38.72 | 2500–2600 | *Pinus tabulaeformis* | T_1_8_ | 1776–1999 | 0.66 | p<0.001 | [118] |
| 198 | Wulan, Qinghai | 98.42 | 36.68 | 3700 | *Sabina przewalskii* | T_6_8_ | 948–2001 | 0.69 | p<0.01 | [119] |
| 199 | nortern Great Khingan | 121.47 | 50.92 | 800–1000 | *Larix gmelini* | T_6_8_ | 1715–2008 | 0.69 | p<0.05 | [120] |
| 200 | Ela Mountain | 98.23 | 36.3 | 3100–3700 | *Sabina tibetica* | T_9_10_ | 140–1993 | 0.60 | p<0.001 | [121] |
| 201 | Wulan, Qinghai | 98.67 | 37.05 | 3910–3964 | *Sabina przewalskii* | T_9_4_ | 1013–2002 | 0.64 | p<0.01 | [122] |
| 202 | Aer Mountain | 120.55 | 47.15 | 1222–1400 | *Larix gmelini* | T_5_9_ | 1822–2008 | 0.63 | p<0.001 | [123] |
| 203 | central Tien Shan | 87.92 | 43.77 | 1970 | *Picea schrenkiana* | PDSI_4_6_ | 1675–2002 | 0.57 | p<0.05 | [124] |
| 204 | central Tien Shan | 88.02 | 43.8 | 2080 | *Picea schrenkiana* | PDSI_4_6_ | 1675–2002 | 0.57 | p<0.05 | [124] |
| 205 | Kaiduhe River Basin | 84.93 | 42.43 | 2300–2410 | *Picea schrenkiana* | T_9_3_ | 1680–2011 | 0.69 | p<0.0001 | [125] |
| 206 | Kaiduhe River Basin | 85.13 | 42.42 | 2437–2730 | *Picea schrenkiana* | T_9_3_ | 1680–2011 | 0.69 | p<0.0001 | [125] |
| 207 | Kayinsayi | 86.42 | 43.57 | 2065–2215 | *Picea schrenkiana* | maxT_5_6_ | 1690–2002 | 0.66 | p<0.0001 | [126] |
| 208 | Kayinsayi | 86.55 | 43.57 | 2475–2615 | *Picea schrenkiana* | maxT_5_6_ | 1690–2002 | 0.66 | p<0.0001 | [126] |
| 209 | Xiaobaidai | 81.25 | 42.75 | 2600–2833 | *Picea schrenkiana* | P_7_6_ | 1770–2005 | 0.74 | p<0.0001 | [127] |
| 210 | Kuerkeeryemie | 81.83 | 42.88 | 2350–2650 | *Picea schrenkiana* | P_7_6_ | 1770–2005 | 0.74 | p<0.0001 | [127] |
| 211 | Kayinsayi | 86.42 | 43.57 | 2065–2215 | *Picea schrenkiana* | P_8_7_ | 1691–2003 | 0.64 | p<0.0001 | [128] |
| 212 | Xirekejiurete | 86.55 | 43.57 | 2475–2615 | *Picea schrenkiana* | P_8_7_ | 1691–2003 | 0.64 | p<0.0001 | [128] |
| 213 | Dashuigou | 81.07 | 44.4 | 1600–1750 | *Picea schrenkiana* | PDSI_1_8_ | 1652–2005 | 0.70 | p<0.05 | [129] |
| 214 | Nilekeyuzan | 82.7 | 44.02 | 1790–1865 | *Picea schrenkiana* | PDSI_1_8_ | 1652–2005 | 0.70 | p<0.05 | [129] |
| 215 | Boerqinggou | 87.12 | 43.13 | 2350–2530 | *Picea schrenkiana* | P_4_5_ | 1535–2001 | 0.65 | p<0.0001 | [130] |
| 216 | Haxionggou | 87.17 | 43.2 | 2370–2640 | *Picea schrenkiana* | P_4_5_ | 1535–2001 | 0.65 | p<0.0001 | [130] |
| 217 | Zhuolesayi | 90.95 | 46.72 | 2422 | *Larix sinirica* | T_6_7_ | 1613–2006 | 0.70 | p<0.0001 | [131] |
| 218 | Bozhou, Xinjiang | 83.25 | 44.38 | 2200–2400 | *Picea schrenkiana* | P_7_6_ | 1622–2010 | 0.60 | p<0.0001 | [132] |
| 219 | Xinyuan, Xinjiang | 84.33 | 43.18 | 2426–2557 | *Picea schrenkiana* | maxT_7_8_ | 1655–2005 | 0.72 | p<0.01 | [133] |
| 220 | Xinyuan, Xinjiang | 84.75 | 43.42 | 2200–2600 | *Picea schrenkiana* | maxT_7_8_ | 1655–2005 | 0.72 | p<0.01 | [133] |
| 221 | Kongnaisi | 84.63 | 43.23 | 2242 | *Picea schrenkiana* | P_7_6_ | 1860–2005 | 0.75 | p<0.0001 | [134] |
| 222 | Altay Mountains | 87.88 | 48.33 | 2310 | *Larix sinirica* | T_6_ | 1570–2005 | 0.64 | p<0.01 | [135] |
| 223 | Hami, Xinjiang | 94.22 | 42.98 | 2894 | *Larix sinirica* | minT_5_9_ | 1800–2005 | 0.55 | p<0.001 | [136] |
| 224 | Shawan, Xinjiang | 84.77 | 44.02 | 2634 | *Picea schrenkiana* | minT_6_8_ | 1650–2001 | 0.59 | p<0.0001 | [137] |
| 225 | Shadawang | 84.63 | 44.08 | 2510–2680 | *Picea schrenkiana* | P_7_8_ | 1624–2002 | 0.75 | p<0.0001 | [138] |
| 226 | Jinghe, Xinjiang | 82.92 | 44.1 | 2370–2650 | *Picea schrenkiana* | T_5_8_ | 1468–2001 | 0.79 | p<0.0001 | [139] |
| 227 | Jinghe, Xinjiang | 83.2 | 44.13 | 2530–2820 | *Picea schrenkiana* | T_5_8_ | 1468–2001 | 0.79 | p<0.0001 | [139] |
| 228 | Urumqi River Source | 87.18 | 43.17 | 2430 | *Picea schrenkiana* | minT_12_3_ | 1542–1994 | 0.61 | p<0.001 | [140] |
| 229 | Xiaganshate, Xinjiang | 87.23 | 43.28 | 2070 | *Picea schrenkiana* | P_5_ | 1667–1993 | 0.77 | p<0.001 | [141] |
| 230 | Zhaosudabaidai | 81.08 | 42.75 | 2270 | *Picea schrenkiana* | P_6_5_ | 1675–1988 | 0.58 | p<0.005 | [142] |
| 231 | Tekesiake | 81.98 | 42.87 | 2110–2724 | *Picea schrenkiana* | P_6_5_ | 1675–1988 | 0.58 | p<0.005 | [142] |
| 232 | Boerqinggou | 87.12 | 43.13 | 2440 | *Picea schrenkiana* | P_7_2_ | 1646–1993 | 0.78 | p<0.001 | [143] |
| 233 | Xibaiyanggou | 87.12 | 43.4 | 2325 | *Picea schrenkiana* | P_7_2_ | 1646–1993 | 0.78 | p<0.001 | [143] |
| 234 | Shakebayidun | 83.65 | 43.65 | 2380 | *Picea schrenkiana* | pP_7_8_ | 1671–2006 | 0.56 | p<0.0001 | [144] |
| 235 | Yingatehe | 79.08 | 41.55 | 2680–2915 | *Picea schrenkiana* | P_8_4_ | 1396–2005 | 0.68 | p<0.0001 | [145] |
| 236 | Zhendanhe | 79.23 | 41.67 | 2910–3170 | *Picea schrenkiana* | T_2_3_ | 1626–2003 | 0.76 | p<0.0001 | [146] |
| 237 | Xiaokuzibayi | 80.38 | 41.78 | 2820–3080 | *Picea schrenkiana* | T_2_3_ | 1626–2003 | 0.76 | p<0.0001 | [146] |
| 238 | Datailan | 80.62 | 41.82 | 2650–3600 | *Picea schrenkiana* | T_2_3_ | 1626–2003 | 0.76 | p<0.0001 | [146] |
| 239 | Aikendaban | 84.8 | 43.2 | 2420–2482 | *Picea schrenkiana* | maxT_5_8_ | 1777–2008 | 0.64 | p<0.001 | [147] |
| 240 | Shaleha | 86.83 | 48.67 | 2220–2320 | *Larix sinirica* | P_6_9_ | 1481–2004 | –0.61 | p<0.0001 | [148] |
| 241 | Fusitangou | 86.47 | 42.42 | 2570–2650 | *Picea schrenkiana* | P_7_6_ | 1360–2004 | 0.73 | p<0.01 | [149] |
| 242 | Jikeyin | 87.65 | 48.55 | 2070–2230 | *Larix sinirica* | T_5_9_ | 1639–2003 | 0.64 | p<0.0001 | [150] |
| 243 | Sanqingshan | 118.05 | 28.9 | 1545 | *Pinus taiwanensis* | maxT_3_10_ | 1806–2009 | 0.75 | p<0.01 | [151] |
| 244 | Songmingyan | 103.4 | 35.24 | 2600 | *Picea  purpurea* | maxT_3_10_ | 1818–2012 | 0.64 | p<0.0001 | [152] |
| 245 | Qamdo | 96.9 | 31.08 | 4320 | *Picea koraiensis* | minT_10_1_ | 1600–2009 | 0.66 | p<0.0001 | [153] |
| 246 | Mt. Shiren | 112.27 | 33.71 | 2010 | *Pinus tabulaeformis* | minT_10_6_ | 1850–2011 | 0.70 | p<0.001 | [154] |
| 247 | Yichang | 111 | 31.02 | 940 | *Pinus  massoniana* | minT_11_4_ | 1875–2011 | 0.74 | p<0.001 | [155] |
| 248 | Bozhou | 82.91 | 40.1 | 2270–2500 | *Picea schrenkiana* | minT_12_11_ | 1494–2010 | 0.59 | p<0.001 | [156] |
| 249 | Tongtianhe | 106.7 | 34.17 | 2150 | *Picea asperata* | minT_12_9_ | 1876–2013 | 0.63 | p<0.001 | [157] |
| 250 | central Qilian | 99.69 | 38.7 | 3400 | *Sabina przewalskii* | minT_1_8_ | 670–2012 | 0.78 | p<0.01 | [158] |
| 251 | Batang | 99.55 | 30.35 | 4160 | *Abies squamata* | minT_6_8_ | 1544–2011 | 0.69 | p<0.001 | [159] |
| 252 | Baiyu | 98.83 | 31.23 | 4012 | *Abies squamata* | minT_6_8_ | 1787–2012 | 0.69 | p<0.001 | [159] |
| 253 | Daofu | 101.25 | 30.68 | 4113 | *Abies squamata* | minT_6_8_ | 1785–2012 | 0.69 | p<0.001 | [159] |
| 254 | Derong | 99.2 | 29.02 | 4221 | *Abies squamata* | minT_6_8_ | 1700–2011 | 0.69 | p<0.001 | [159] |
| 255 | Seda | 100.83 | 31.83 | 3671 | *Abies squamata* | minT_6_8_ | 1798–2012 | 0.69 | p<0.001 | [159] |
| 256 | Miyaluo | 102.23 | 31.63 | 3750 | *Sabina squamata* | minT_6_8_ | 1785–2012 | 0.69 | p<0.001 | [159] |
| 257 | Xinlong | 100.17 | 30.73 | 3522 | *Abies squamata* | minT_6_8_ | 1764–2012 | 0.69 | p<0.001 | [159] |
| 258 | Alihe | 124.47 | 50.64 | 376 | *Pinus sylvestris* | P_12_1_ | 1809–2009 | 0.68 | p<0.001 | [160] |
| 259 | Mt. Xianxia | 119.45 | 28.6 | 380–490 | *Pinus massoniana* | P_2_4_ | 1856–2013 | 0.69 | p<0.01 | [161] |
| 260 | Mt. Xinglong | 104.05 | 35.78 | 2450 | *Picea wilsonii* | P_7_5_ | 1816–2010 | 0.75 | p<0.0001 | [162] |
| 261 | Nanmulin | 89.11 | 30.08 | 4500–4700 | *Sabina tibetica* | P_7_6_ | 1485–2010 | 0.68 | p<0.001 | [163] |
| 262 | Talidesayi | 89 | 47.8 | 1217 | *Picea obovata* | P_7_6_ | 1722–2012 | 0.74 | p<0.001 | [164] |
| 263 | Daqiao | 89.64 | 47.42 | 1645 | *Picea obovata* | P_7_6_ | 1722–2012 | 0.74 | p<0.001 | [164] |
| 264 | Fuyun | 88.8 | 47.7 | 1150–1700 | *Siberian spruce* | P_7_6_ | 1825–2010 | 0.81 | p<0.01 | [165] |
| 265 | Mohe | 120.79 | 52.75 | 590 | *Pinus sylvestris* | P_8_7_ | 1724–2008 | 0.63 | p<0.0001 | [166] |
| 266 | Songmingyan | 103.39 | 35.23 | 2589 | *Sabina przewalskii* | P_8_7_ | 1773–2010 | 0.56 | p<0.01 | [167] |
| 267 | Mt. Shennong | 112.8 | 35.25 | 986 | *Pinus bungeana* | P_9_6_ | 1850–2010 | 0.65 | p<0.0001 | [168] |
| 268 | Mt. Shennong | 112.79 | 35.22 | 990 | *Pinus bungeana* | PDSI_11_10_ | 1805–2005 | 0.59 | p<0.0001 | [169] |
| 269 | Mt. Huanglong | 109.5 | 39.5 | 1130–1320 | *Pinus tabulaeformis* | PDSI_1_7_ | 1760–2010 | 0.64 | p<0.01 | [170] |
| 270 | Lamadong | 111.28 | 40.78 | 1300 | *Pinus tabulaeformis* | PDSI_3_6_ | 1680–2012 | 0.64 | p<0.0001 | [171] |
| 271 | Mt. Lingkong Mountain | 112.08 | 36.6 | 1480–1700 | *Pinus tabulaeformis* | PDSI_3_8_ | 1703–2008 | 0.68 | p<0.001 | [172] |
| 272 | western Qilian | 98.87 | 39.61 | 3350 | *Sabina przewalskii* | PDSI_5_7_ | 1161–2010 | 0.63 | p<0.01 | [173] |
| 273 | Mt. Hengshan | 113.72 | 39.68 | 1750 | *Pinus tabulaeformis* | PDSI_5_7_ | 1767–2012 | 0.73 | p<0.01 | [174] |
| 274 | Mt. Xiaowutai | 114.69 | 39.75 | 1550 | *Pinus tabulaeformis* | PDSI_5_7_ | 1767–2012 | 0.73 | p<0.01 | [174] |
| 275 | Mt.Guancen | 112.08 | 38.83 | 1600–2100 | *Pinus tabulaeformis* | PDSI_6_7_ | 1810–2003 | 0.69 | p<0.0001 | [175] |
| 276 | central Fujian | 117.5 | 25.92 | 998 | *Toona sinensis* | PDSI_7_2_ | 1855–2011 | 0.60 | p<0.001 | [176] |
| 277 | central Fujian | 117.7 | 27.8 | 950 | *Toona sinensis* | PDSI_7_2_ | 1855–2011 | 0.60 | p<0.001 | [176] |
| 278 | Baluntai, Xinjiang | 86.48 | 42.6 | 2800 | *Picea schrenkiana* | P_7_6_ | 1464–2005 | 0.72 | p<0.0001 | [177] |
| 279 | Mt. Wolong | 102.99 | 30.85 | 3700 | *Sabina saltuaria* | T_10_9_ | 1840–2011 | 0.74 | p<0.01 | [178] |
| 280 | Shennongjia | 110.28 | 31.48 | 2750 | *Abies fargesii* | T_2_5_ | 1783–2011 | 0.66 | p<0.001 | [179] |
| 281 | Caolianling | 109.83 | 34.3 | 2600 | *Abies chensiensis* | T_2_6_ | 1870–2012 | 0.61 | p<0.01 | [180] |
| 282 | Zhouqu | 103.8 | 34.12 | 3120–3200 | *Abies faxoniana* | T_2_7_ | 1650–2006 | 0.66 | p<0.001 | [181] |
| 283 | Mt. Shimen | 106.15 | 34.45 | 2100 | *Pinus tabulaeformis* | T_5_7_ | 1630–2011 | –0.70 | p<0.0001 | [182] |
| 284 | Mt.Wudao | 113.58 | 28.18 | 200–350 | *Pinus  massoniana* | T_6_9_ | 1815–2013 | –0.52 | p<0.01 | [183] |
| 285 | Songpan | 103.5 | 32.65 | 3300–3500 | *Abies faxoniana* | T_9_8_ | 1701–2010 | 0.58 | p<0.001 | [184] |
| 286 | Delingha | 97.37 | 37.37 | 3820 | *Juniperus spp.* | PDSI_5_6_ | 976–2000 | 0.66 | p<0.001 | [185] |
| 287 | Wulan | 98.55 | 36.94 | 3640 | *Juniperus spp.* | PDSI_5_6_ | 1322–2001 | 0.66 | p<0.001 | [185] |
| 288 | Dulan | 98.13 | 36.37 | 3610 | *Juniperus spp.* | PDSI_5_6_ | –2278 | 0.66 | p<0.001 | [185] |
| 289 | Tongren | 102.02 | 35.83 | 3260 | *Juniperus spp.* | PDSI_5_6_ | 1630–2001 | 0.66 | p<0.001 | [185] |
| 290 | Zhongtie | 100.1 | 35.05 | 3831 | *Juniperus spp.* | PDSI_5_6_ | 1470–2008 | 0.66 | p<0.001 | [185] |
| 291 | Jiangqun | 100.35 | 35.02 | 3626 | *Juniperus spp.* | PDSI_5_6_ | 1465–2005 | 0.66 | p<0.001 | [185] |
| 292 | Xueshan | 99.84 | 34.8 | 3644 | *Juniperus spp.* | PDSI_5_6_ | 1320–2005 | 0.66 | p<0.001 | [185] |
| 293 | Hebei | 100.81 | 34.76 | 3320 | *Juniperus spp.* | PDSI_5_6_ | 1442–2005 | 0.66 | p<0.001 | [185] |
| 294 | Ningmute | 101 | 34.62 | 3575 | *Juniperus spp.* | PDSI_5_6_ | 1506–2008 | 0.66 | p<0.001 | [185] |
| 295 | Dongzhong | 97.67 | 32.52 | 4018 | *Juniperus spp.* | PDSI_5_6_ | 1396–2001 | 0.66 | p<0.001 | [185] |
| 296 | Dongba | 95.64 | 32.19 | 4192 | *Juniperus spp.* | PDSI_5_6_ | 1515–2001 | 0.72 | p<0.001 | [185] |
| 297 | Jiangxi | 97.07 | 32.07 | 3637 | *Juniperus spp.* | PDSI_5_6_ | 1515–2001 | 0.72 | p<0.001 | [185] |
| 298 | Baizha | 96.52 | 31.87 | 3908 | *Juniperus spp.* | PDSI_5_6_ | 1378–2001 | 0.72 | p<0.001 | [185] |
| 299 | Suoxian | 94.29 | 31.63 | 3854 | *Juniperus spp.* | PDSI_5_6_ | 1753–2004 | 0.72 | p<0.001 | [185] |
| 300 | Biru | 93.87 | 31.12 | 4350 | *Juniperus spp.* | PDSI_5_6_ | 1475–2005 | 0.72 | p<0.001 | [185] |
| 301 | Bianbamx | 94.58 | 31.08 | 4144 | *Juniperus spp.* | PDSI_5_6_ | 1449–2006 | 0.72 | p<0.001 | [185] |
| 302 | Gongjue | 98.69 | 30.75 | 3817 | *Juniperus spp.* | PDSI_5_6_ | 1475–2006 | 0.72 | p<0.001 | [185] |
| 303 | Jiali | 93.46 | 30.6 | 4250 | *Juniperus spp.* | PDSI_5_6_ | 1542–2004 | 0.72 | p<0.001 | [185] |
| 304 | Luolong | 96.18 | 30.58 | 4440 | *Juniperus spp.* | PDSI_5_6_ | 1548–2006 | 0.72 | p<0.001 | [185] |
| 305 | Linzhou | 91.51 | 30.31 | 4233 | *Juniperus spp.* | PDSI_5_6_ | 1442–2004 | 0.72 | p<0.001 | [185] |
| 306 | Basu | 97.12 | 30.06 | 4382 | *Juniperus spp.* | PDSI_5_6_ | 1702–2006 | 0.72 | p<0.001 | [185] |
| 307 | Gbjda | 92.65 | 29.82 | 4250 | *Juniperus spp.* | PDSI_5_6_ | 1611–2004 | 0.72 | p<0.001 | [185] |
| 308 | Mangkang | 98.35 | 29.45 | 4050 | *Juniperus spp.* | PDSI_5_6_ | 1451–2006 | 0.72 | p<0.001 | [185] |

In this table, climatic factor represents the reconstructed climate element using tree–ring width chronology in each site. PDSI represents the Palmer drought severity index, P represents precipitation, T represents temperature, maxT represents maximum temperature, and minT represents minimum temperature. The ‘p’ before them represents the previous year of tree growth. The subscripting numbers after them represent months (the numbers from 1–12 are months from January–December, respectively).

The maxT_11_2_ represents maximum temperature of previous November to current February.

The maxT_1_4_ represents maximum temperature of January to April of current year.

The maxT_5_7_ represents maximum temperature of May to July of current year.

The maxT_6_ represents maximum temperature of June of current year.

The maxT_7_ represents maximum temperature of July of current year.

The maxT_4_6_ represents maximum temperature of April to June of current year.

The maxT_4_9_ represents maximum temperature of April to September of current year.

The maxT_5_6_ represents maximum temperature of May to June of current year.

The maxT_6_9_ represents maximum temperature of June to September of current year.

The maxT_7_8_ represents maximum temperature of July to August of current year.

The maxT_5_8_ represents maximum temperature of May to August of current year.

The maxT_3_10_ represents maximum temperature of March to October of current year.

The minT_10_6_ represents minimum temperature of previous October to current June.

The minT_11_4_ represents minimum temperature of previous November to current April.

The minT_12_11_ represents minimum temperature of previous December to current November.

The minT_12_9_ represents minimum temperature of previous December to current September.

The minT_1_8_ represents minimum temperature of January to August of current year.

The minT_6_8_ represents minimum temperature of June to August of current year.

The minT_10_1_ represents minimum temperature of previous October to current January.

The minT_10_4_ represents minimum temperature of previous October to current April.

The minT_6_8_ represents minimum temperature of June to August of current year.

The minT_12_3_ represents minimum temperature of previous December to current March.

The minT_5_9_ represents minimum temperature of May to September of current year.

The minT_10_9_ represents minimum temperature of previous October to current September.

The minT_11_3_ represents minimum temperature of previous November to current March.

The minT_1_4_ represents minimum temperature of January to April of current year.

The minT_1_7_ represents minimum temperature of January to July of current year.

The minT_6_7_ represents minimum temperature of June to July of current year.

The minT_7_10_ represents minimum temperature of July to October of current year.

The minT_8_ represents minimum temperature of August of current year.

The T_2_5_ represents temperature of February to May of current year.

The T_2_6_ represents temperature of February to June of current year.

The T_6_9_ represents temperature of June to September of current year.

The T_9_8_ represents temperature of previous September to current August.

The T_10_9_ represents temperature of previous October to current September.

The T_12_3_ represents temperature of previous December to current March.

The T_12_4_ represents temperature of previous December to current April.

The T_1_4_ represents temperature of January to April of current year.

The T_1_7_ represents temperature of January to July of current year.

The T_2_4_ represents temperature of February to April of current year.

The T_2_7_ represents temperature of February to July of current year.

The T_3_4_ represents temperature of March to April of current year.

The T_4_9_ represents temperature of April to September of current year.

The T_5_6_ represents temperature of May to June of current year.

The T_5_7_ represents temperature of May to July of current year.

The T_5_8_ represents temperature of May to August of current year.

The T_6_7_ represents temperature of June to July of current year.

The T_6_8_ represents temperature of June to August of current year.

The T_7_ represents temperature of July of current year.

The T_9_10_ represents temperature of September to October of current year.

The T_9_4_ represents temperature of previous September to current April.

The T_1_3_ represents temperature of January to March of current year.

The pT_10_ represents temperature of October of previous year.

The T_1_6_ represents temperature of January to June of current year.

The T_2_9_ represents temperature of February to September of current year.

The T_7_6_ represents temperature of previous July to current June.

The pT_1_12_ represents temperature of January to December of previous year.

The T_1_8_ represents temperature of January to August of current year.

The T_5_9_ represents temperature of May to September of current year.

The T_9_3_ represents temperature of previous September to current March.

The T_6_ represents temperature of June of current year.

The T_2_3_ represents temperature of February to March of current year.

The P_9_6_ represents precipitation of previous September to current June.

The P_2_3_ represents precipitation of February to March of current year.

The P_2_5_ represents precipitation of February to May of current year.

The P_4_7_ represents precipitation of April to July of current year.

The P_7_6_ represents precipitation of previous July to current June.

The P_8_7_ represents precipitation of previous August to current July.

The P_9_7_ represents precipitation of previous September to current July.

The P_1_7_ represents precipitation of January to July of current year.

The P_2_7_ represents precipitation of February to July of current year.

The P_3_4_ represents precipitation of March to April of current year.

The P_3_5_ represents precipitation of March to May of current year.

The P_5_7_ represents precipitation of May to July of current year.

The P_4-5_ represents precipitation of April to May of current year.

The P_6_ represents precipitation of June of current year.

The P_8_6_ represents precipitation of previous August to current June.

The P_9_8_ represents precipitation of previous September to current August.

The P_7_8_ represents precipitation of July to August of current year.

The P_5_ represents precipitation of May of current year.

The P_6-5_ represents precipitation of previous June to current May.

The P_7-2_ represents precipitation of previous July to current February.

The pP_7_8_ represents precipitation of July to August of previous year.

The P_8_4_ represents precipitation of previous August to current April.

The P_6_9_ represents precipitation of June to September of current year.

The P_12-1_ represents precipitation of previous December to current January.

The P_7-5_ represents precipitation of previous July to current May.

The P_2-4_ represents precipitation of February to April of current year.

The PDSI_11_1_ represents PDSI of previous November to current January.

The PDSI_3_5_ represents PDSI of March to May of current year.

The PDSI_5_4_ represents PDSI of previous May to current April.

The PDSI_3_6_ represents PDSI of March to June of current year.

The PDSI_5_6_ represents PDSI of May to June of current year.

The PDSI_5_7_ represents PDSI of May to July of current year.

The PDSI_5_8_ represents PDSI of May to August of current year.

The PDSI_8_7_ represents PDSI of previous August to current July.

The PDSI_4_7_ represents PDSI of April to July of current year.

The PDSI_3_7_ represents PDSI of March to July of current year.

The PDSI_3_9_ represents PDSI of March to September of current year.

The PDSI_9_8_ represents PDSI of September to August of current year.

The PDSI_4_6_ represents PDSI of April to June of current year.

The PDSI_1_8_ represents PDSI of January to August of current year.

The PDSI_11_10_ represents PDSI of previous November to current October.

The PDSI_1_7_ represents PDSI of January to July of current year.

The PDSI_3_8_ represents PDSI of March to August of current year.

The PDSI_6_7_ represents PDSI of June to July of current year.

The PDSI_7-2_ represents PDSI of previous July to current February.

**References:**

1. Chen F, Yuan YJ and Wei WS *et al.* Tree ring-based winter temperature reconstruction for Changting Fujian subtropical region of Southeast China since 1850: linkages to the Pacific Ocean. *Theor Appl Climatol* 2012; **109:** 141–151.
2. Shao XM and Wu XD. Reconstruction of climate change on Changbai Mountain Northeast China using tree-ring data. *Quatern Sci* 1997; **1:** 76–85.
3. Liu Y, Wang YC and Li Q *et al.* Reconstructed May–July mean maximum temperature since 1745 AD based on tree–ring width of Pinus tabulaeformis in Qianshan Mountain China. *Palaeogeogr Palaeocl Palaeoec* 2013; **388:** 145–152.
4. Tian QH, Liu Y and Cai QF *et al.* The Maximum Temperature of May–July Inferred from Tree-ring in Funiu Mountain since 1874 AD. *Acta Geogr Sin* 2009; **64:** 879–887.
5. Qin NS, Shi XH and Shao XM *et al.* Average maximum temperature change recorded by tree rings in West Sichuan Plateau. *Plat MT Meterol Res* 2008; **28:** 18–24.
6. Xiao DM, Qin NS and Li JJ *et al.* Change of mean maximum temperature in July during 1506–2008 in Seda of West Sichuan Plateau according to reconstructed tree-ring series.*JDesert Res* 2013; **33:** 1536–1543.
7. Liu Y, Lei Y and Song HM *et al.* The annual mean lowest temperature reconstruction based on Pinus Bungeanas (Pinus bungeana Zucc) ring width in the Yulin Region Shandong China since AD 1616. *J Earth Environ* 2010; **1:** 28–35.
8. Song HM, Liu Y and Ni WM *et al.* Winter mean lowest temperature derived from tree-ring width in Jiuzhaigou region China since 1750 AD. *Quatern Sci* 2007; **27:** 486–491.
9. Shao XM and Fan JM. Past climate on West Sichuan Plateau as reconstructed from ring-widths of dragon spruce. *Quatern Sci*1999; **1:** 81–89.
10. Shi JF, Cook ER and Li JB *et al.* Unprecedented January–July warming recorded in a 178–year tree-ring width chronology in the Dabie Mountains southeastern China. *Palaeogeogr Palaeocl Palaeoec* 2013; **381:** 92–97.
11. Yu SL, Yuan YJ and Wei WS *et al.* Reconstruction of minimum temperature field in June–July during 1787–2005 in the West Sichuan Plateau. *J Desert Res*2012; **32:** 1010–1016.
12. Chen F, Yuan YJ and Wei WS *et al.* Reconstructed temperature for Yong'an Fujian Southeast China: Linkages to the Pacific Ocean climate variability. *Global Planet Change* 2012; **86–87:** 11–19.
13. Zhu HF, Shao XM and Yin ZY *et al.* August temperature variability in the southeastern Tibetan Plateau since AD 1385 inferred from tree rings. *Palaeogeogr Palaeocl Palaeoec* 2011; **305:** 84–92.
14. Gou XH, Yang T and Gao LL *et al.* A 457–year reconstruction of precipitation in the southeastern Qinghai–Tibet Plateau China using tree-ring records. *Chin Sci Bull* 2013; **58:** 1107–1114.
15. Li ZS, Shi C and Liu Y*et al.* Winter drought variations based on tree-ring data in Gaoligong Mountain northwestern Yunnan China A D 1795–2004.*Pakistan J Bot* 2011; **43:** 2469–2478.
16. Fan ZX, Brauning A and Cao KF Tree-ring based drought reconstruction in the central Hengduan Mountains region(China) since AD 1655. *Int J Climatol* 2008; **28:** 1879–1887.
17. Fang KY, Gou XH and Chen FH *et al.* Reconstructed droughts for the southeastern Tibetan Plateau over the past 568years and its linkages to the Pacific and Atlantic Ocean climate variability. *Clim Dynam* 2010; **35:** 577–585.
18. Fan ZX, Brauning A and Cao KF. Annual temperature reconstruction in the central Hengduan Mountains China as deduced from tree rings. *Dendrochronologia* 2008; **26:** 97–107.
19. Shi JF, Cook ER and Lu HY *et al.* Tree-ring based winter temperature reconstruction for the lower reaches of the Yangtze River in southeast China. *Clim Res* 2010; **41:** 169–175.
20. Shi J, Lu H and Wan J *et al.* Winter–half year temperature reconstruction of the last century using Pinus armandii Franch tree-ring width chronologies in the eastern Qinling Mountains. *Quatern Sci*2009; **29:** 831–836.
21. Liu HB and Shao XM. Reconstruction of early–spring temperature of Qinling Mountains using tree-ring chronologies. *Acta Geogr Sin* 2003; **58:** 879–884.
22. Duan JP, Zhang QB and Lv LX *et al.* Regional-scale winter–spring temperature variability and chilling damage dynamics over the past two centuries in southeastern China. *Clim Dynam* 2012; **39:** 919–928.
23. Liu Y, Liu N and Song HM *et al.* Reconstructed mean air temperature from January to July at the divide sampling site in the mid–Qinling Mountains with tree-ring widths. *Adv Clim Change Res*2009; **5:** 260–265.
24. Zhu HF, Fang XQ and Shao XM *et al.* Tree ring-based February–April temperature reconstruction for Changbai Mountain in Northeast China and its implication for East Asian winter monsoon. *Clim Past* 2009; **5:** 661–666.
25. Zheng YH, Zhang Y and Shao XM *et al.* Temperature variability inferred from tree-ring widths in the Dabie Mountains of subtropical central China. *Trees–Struct Funct* 2012; **26:** 1887–1894.
26. Liu Y, Ma LM and Hughes MK *et al.* Seasonal temperature reconstruction from Central China based on Tree-Ring data.*Palaeobotanist*2001; **50:** 89–94.
27. Cai QF, Liu Y and Song HM *et al.* Tree-ring-based reconstruction of the April to September mean temperature since 1826AD for north–central Shaanxi Province China. *Sci China Ser D* 2008; **51:** 1099–1106.
28. Liu Y, Tian QH and Song HM *et al.* Tree-ring width based May–June mean temperature reconstruction for Huashan Mountains since AD 1558and 20th century warming. *Quatern Sci* 2009; **29:** 888–895.
29. Liu Y, Linderholm HM and Song HM *et al.* Temperature variations recorded in Pinus tabulaeformis tree rings from the southern and northern slopes of the central Qinling Mountains central China. *Boreas* 2009; **38:** 285–291.
30. Fan ZX, Brauning A and Tian Q H *et al.* Tree ring recorded May–August temperature variations since AD 1585in the Gaoligong Mountains southeastern Tibetan Plateau. *Palaeogeogr Palaeocl Palaeoec* 2010; **296:** 94–102.
31. Li ZS, Liu G and Fu BJ *et al.* Tree ring-based summer temperature reconstruction over the past 200 years in Miyaluo of western Sichuan China. *Quatern Sci* 2011; **31:** 522–534.
32. Li ZS, Liu GH and Zhang QB *et al.* Tree ring reconstruction of summer temperature variations over the past 159years in Wolong National Natural Reserve western Sichuan China. *Chin J Plant Ecol* 2010; **34:** 628–641.
33. Liang EY, Shao XM and Xu Y. Tree-ring evidence of recent abnormal warming on the southeast Tibetan Plateau. *Theor Appl Climatol* 2009; **98:** 9–18.
34. Li ZS, Shi CM and Liu Y *et al.* Summer mean temperature variation from 1710–2005inferred from tree-ring data of the Baimang Snow Mountains northwestern Yunnan China. *Clim Res* 2011; **47:** 207–218.
35. Li ZS, Zhang QB and Ma KP. Tree-ring reconstruction of summer temperature for AD 1475–2003in the central Hengduan Mountains Northwestern Yunnan China. *Clim Change*2012; **110:** 455–467.
36. Yu SL, Yuan YJ and Wei WS *et al.* Reconstructed mean temperature in Mearkang West Sichuan in July and its detection of climatic period signal. *Plateau Meteorol* 2012; **31:** 193–200.
37. Wang WW, Zhang JH and Dai GH *et al.* Variation of autumn temperature over the past 240years in Changbai Mountains of Northeast China: A reconstruction with tree-ring records. *Chin J Ecol* 2012; **31:** 787–793.
38. Duan JP, Zhang QB and Lv LX. Increased variability in cold–season temperature since the 1930s in subtropical China. *J Clim* 2013; **26:** 4749–4757.
39. Qin NS, Shao XM and Shi XH *et al.* Tree-ring chronology in southern Qinghai and its relation to climatic element. *Plateau Meteorol* 2003; **22:** 445–450.
40. Gou XH, Yang MX and Peng JF *et al.* Maximum temperature reconstruction for Animaqing Mountains over past 830 years based on tree-ring records. *Quatern Sci*2006; **26:** 991–998.
41. Gou XH, Peng JF and Chen FH *et al.* A dendrochronological analysis of maximum summer half–year temperature variations over the past 700 years on the northeastern Tibetan Plateau. *Theor Appl Climatol* 2008; **93:** 195–206.
42. Shi XH, Qin NS and Zhu HF *et al.* May–June mean maximum temperature change during 1360–2005 As reconstructed by tree rings of Sabina Tibetica in Zaduo Qinghai Province. *Chin Sci Bull* 2010; **55:** 3023–3029.
43. Zhang RB, Yuan YJ and Wei WS *et al.* Analysis on mean minimum temperature in the East Tibet from Autumn to winter in tree ring of the past 400years. *Plateau Meteorol* **29:** 359–365. 2010;
44. Gou XH, Chen FH and Jacoby G *et al.* Rapid tree growth with respect to the last 400 years in response to climate warming northeastern Tibetan Plateau. *Int J Climatol* 2007; **27:** 1497–1503.
45. Liang EY, Shao XM and Qin NS. Tree-ring based summer temperature reconstruction for the source region of the Yangtze River on the Tibetan Plateau. *Global Planet Change*2008; **61:** 313–320.
46. Cai QF, Liu Y and Yang YK *et al.* The reconstruction of tree-ring chronology and early spring (from February to March) precipitation information in Huanglong region Shaanxi province. *Mar Geol Quatern Geol* 2005; **25:** 133–139.
47. Wei BY, Li YN and Yan XD *et al.* Estimation of February–May precipitations from tree-ring in Xiaowutai Mountain region from 1895 onwards. *J Beijing Normal Univ* 2008; **44:** 96–102.
48. Fang KY, Gou XH and Chen FH *et al.* Precipitation variability during the past 400 years in the Xiaolong Mountain. central China inferred from tree rings. *Clim Dynam* 2012; **39:** 1697–1707.
49. Liu JJ, Yang Band Qin C. Tree-ring based annual precipitation reconstruction since AD 1480 in south central Tibet. *Quatern Int* 2011; **236:** 75–81.
50. He MH, Yang B and Brauning A  *et al.* Tree-ring derived millennial precipitation record for the south–central Tibetan Plateau and its possible driving mechanism. *Holocene* 2013; **23:** 36–45.
51. Liu Y, Sun B and Song HM *et al.* Tree-ring-based precipitation reconstruction for Mt Xinglong China since AD 1679. *Quatern Int* 2013; **283:** 46–54.
52. Liu CG, Gou XH and Fang KY *et al.* Precipitation reconstruction in southern Gansu province since AD 1824. *Quatern Sci* 2013; **33:** 518–525.
53. Liu Y, Tian H and Song HM *et al.* Tree ring precipitation reconstruction in the Chifeng–Weichang region China and East Asian summer monsoon variation since AD 1777. *J Geophys Res Atmos* 2010; **115:** D06103.
54. Li Q, Liu Y and Song HM *et al.* Reconstruction of annual precipitation since 1686 AD from Ningwu region Shanxi province. *Quatern Sci* 2006; **26:** 999–1006.
55. Wang XC, Zhang QB and Ma KP *et al.* A tree-ring record of 500–year dry–wet changes in northern Tibet China. *Holocene* 2008; **18:** 579–588.
56. Cai QF and Liu Y. Climatic response of Chinese pine and PDSI variability in the middle Taihang Mountains north China since 1873. *Trees–Struct Funct* 2013; **27:** 419–427.
57. Song HM and Liu Y. PDSI variations at Kongtong Mountain China inferred from a 283–year Pinus tabulaeformis ring width chronology. *J Geophys Res Atmos* 2011; **116:** D22111.
58. Fang KY, Gou XH and Chen FH *et al.* Tree-ring based reconstruction of drought variability. 1615–2009; in the Kongtong Mountain area northern China. *Global Planet Change* 2012; **80–81:** 190–197.
59. Fang KY, Gou XH and Chen FH  *et al.* Tree-ring based drought reconstruction for the Guiqing Mountain (China): linkages to the Indian and Pacific Oceans. *Int J Climatol* 2010; **30:** 1137–1145.
60. Fang KY, Gou XH and Chen FH *et al.* Drought variations in the eastern part of northwest China over the past two centuries: evidence from tree rings. *Clim Res* 2009; **38:** 129–135.
61. Yin H, Liu H and Huang L *et al.* Reconstruction of October mean temperature since 1796 in Wuying based on tree ring data. *Adv Clim Change Res* 2010; **1:** 100–106.
62. Sun JY, Liu Y and Sun B *et al.* Tree-ring based PDSI reconstruction since 1853 AD in the source of the Fenhe river basin Shanxi province China. *Sci China Earth Sci* 2012; **55:** 1847–1854.
63. Yang B, Kang XC and Bräuning A *et al.* A 622–year regional temperature history of southeast Tibet derived from tree rings. *Holocene*2010; **20:** 181–190.
64. Song HM, Liu Y and Li Q *et al.* Tree-ring derived temperature records in the central Loess Plateau China. *Quatern Int* 2013; **283:** 30–35.
65. Zhu HF, Shao XM and Yin ZY *et al.* Early summer temperature reconstruction in the eastern Tibetan plateau since ad 1440: using tree-ring width of Sabina tibetica. *Theor Appl Climatol* 2011; **106:** 45–53.
66. Cai QF, Liu Y and Tian H. A dendroclimatic reconstruction of May–June mean temperature variation in the Heng Mountains north China since 1767 AD .*Quatern Int* 2013; **283:** 3–10.
67. Liu Y, Tian H and Song HM *et al.* Tree ring based reconstruction of the May–June mean temperature since AD 1884 in Weichang Hebei Province China. *Quatern Sci* 2009; **29:** 896–904.
68. Cai QF, Liu Y and Bao G *et al.* Tree-ring-based May–July mean temperature history for Luliang Mountains China since 1836 .*Chin Sci Bull* 2010; **55:** 3008–3014.
69. Li Q, Liu Y and Song HM *et al.* Long-term variation of temperature over North China and its links with large-scale atmospheric circulation .*Quatern Int* 2013; **283:** 11–20.
70. Hou Y, Wang N and Li G *et al.* Reconstruction of summer average temperature from tree-ring proxy data during 1751–2005 in Mt. Kongtong. *Adv Clim Change Res* 2007; **3:** 172–176.
71. Yang B, Kang XC and Liu JJ *et al.* Annual temperature history in Southwest Tibet during the last 400 years recorded by tree rings. *Int J Climatol*2010; **30:** 962–971.
72. Peng JJ, Sun Y and Chen M *et al.* Tree-ring based precipitation variability since AD 1828 in northwestern Liaoning China. *Quatern Int* 2013; **283:** 63–71.
73. Bao G, Liu Y and Linderholm HW. April–September mean maximum temperature inferred from Hailar pine. Pinus sylvestris var mongolica; tree rings in the Hulunbuir region Inner Mongolia back to 1868 AD. *Palaeogeogr Palaeocl* 2012; **313:** 162–172.
74. Zhu XD, Qing NS and Li DL *et al.* May–June surface temperatures of Chaidamu Basin reestablished using tree-ring. Chronology *Plateau Meteorol* 2005; **24:** 331–337.
75. Chen F, Yuan YJ and Wei WS *et al.* Temperature change recorded by tree ring in Jiuquan during the period from June to September in recent 240 years. *Arid Zone Res* 2012; **29:** 47–54.
76. Liu Y, Cai QF and Shi JF *et al.* Seasonal precipitation in the south–central Helan Mountain region China reconstructed from tree-ring width for the past 224 years. *Can J Forest Res* 2005; **35:** 2403–2412.
77. Shi JF, Liu Y and Cai QF *et al.* A196–year precipitation reconstruction based on tree-ring width in the Helan Mountains of northern China and the precipitation variability. *Mar Geol Quatern Geol* 2007; **27:** 96–101.
78. Liu Y, Sun JY and Yang YK *et al.* Tree-ring-derived precipitation records from inner Mongolia China since ad 1627.*Tree-ring Res* 2007; **63:** 3–14.
79. Gou XH, Chen FH and Wang YJ *et al.* Spring precipitation reconstructed in the East of the Qilian Mountain during the last 280 years by tree ring width. *J Glaciol Geocryol* 2001; **23:** 292–296.
80. Wang YJ, Chen FH and Gou XH. Reconstruction of spring precipitation in the middle region of the Qilian Mountains using tree-ring data. *Sci Geograph Sin* 2001; **21:** 373–377.
81. Liu Y, Cai QF and Park WK *et al.* Tree-ring precipitation records from Baiyinaobao Inner Mongolia since AD 1838. *Chin Sci Bull* 2003; **48:** 1140–1145.
82. Fan ZA , Wei WS and Chen F *et al .*Precipitation variation from 1775to 2005 at the eastern margin of Tengger Desert China inferred from tree-ring. *J Desert Res* 2012; **32:** 996–1002.
83. Liu Y, Shi JF and Shishov V *et al.* Reconstruction of May–July precipitation in the north Helan Mountain Inner Mongolia since AD 1726from tree-ring late-wood widths. *Chin Sci Bull* 2004; **49:** 405–409.
84. Ma LM, Liu Y and Cai QF *et al.* The precipitation records from tree-ring latewood width in the Helan Mountain. *Mar Geol Quatern Geol* 2003; **23:** 109–114.
85. Liu WH, Gou XH and Yang MX *et al.* Drought reconstruction in the Qilian Mountains over the last two centuries and its implications for large-scale moisture patterns. *Adv Atmos Sci* 2009; **26:** 621–629.
86. Yang B, Qin C and Bräuning A *et al.* Rainfall history for the Hexi Corridor in the arid northwest China during the past 620 years derived from tree rings .*Int J Climatol* 2011; **31:** 1166–1176.
87. Liang EY, Shao XM and Eckstein D *et al .*Topography– and species-dependent growth responses of Sabina przewalskii and Picea crassifolia to climate on the northeast Tibetan Plateau. *Forest Ecol Manag* 2006; **236:** 268–277.
88. Liu Y, Lei Y and Sun B *et al.* Annual precipitation variability inferred from tree-ring width chronologies in the Changling–Shoulu region China during AD 1853–2007.*Dendrochronologia* 2013; **31:** 290–296.
89. Yang YK, Liu Y and Cai QF *et al.* Precipitation reconstruction from tree ring width over the central Qilian Mountains for the last 248 years. *Mar Geol Quatern Geol* 2005; **25:** 113–118.
90. Chen F, Yuan YJ and Wei WS. Climatic response of Picea crassifolia tree-ring parameters and precipitation reconstruction in the western Qilian Mountains China. *J Arid Environ*2011; **75:** 1121–1128.
91. Liu Y, Sun JY and Song HM *et al.* Tree-ring hydrologic reconstructions for the Heihe River watershed western China since AD 1430.*Water Res* 2010; **44:** 2781–2792.
92. Sun JY and Liu Y. Tree ring based precipitation reconstruction in the south slope of the middle Qilian Mountains northeastern Tibetan Plateau over the last millennium. *J Geophys Res Atmos*2012; **117:** D08108.
93. Liu Y, An ZS and Ma HZ *et al.* Precipitation variation in the northeastern Tibetan Plateau recorded by the tree rings since 850 AD and its relevance to the Northern Hemisphere temperature. *Sci China Ser D* 2006; **49:** 77–91.
94. Liu Y, Bao G and Song HM  *et al.* Precipitation reconstruction from Hailar pine (Pinus sylvestris var mongolica) tree rings in the Hailar region Inner Mongolia China back to 1865AD. *Palaeogeogr Palaeocl Palaeoec* 2009; **282:** 81–87.
95. Shao XM. Reconstruction of precipitation variation from tree rings in recent 1000 years in Delingha Qinghai. *Sci China Ser D* 2005; **48:** 939–949.
96. Shao XM, Liang EY and Huang L *et al.* A reconstructed precipitation series over the past millennium in the northeastern Qaidam Basin. *Adv Clim Change Res* 2006; **2:** 122–126.
97. Chen F, Yuan YJ and Wei WS *et al.* Reconstruction of annual precipitation in Shandan based tree-ring since 1783 AD. *Geogr Geo-Informa Sci*2010; **26:** 82–86.
98. Li YJ, Gou XH and Fang KY *et al.* Reconstruction of precipitation of previous August to current June during 1821–2008in the eastern Qilian Mountains. *J Desert Res* 2012; **32:** 1393–1401.
99. Tian QH, Zhou XJ and Gou XH *et al.* Analysis of reconstructed annual precipitation from tree-rings for the past 500 years in the middle Qilian Mountain. *Sci China Earth Sci* 2012; **55:** 770–778.
100. Zhang Y, Tian QH and Gou XH *et al.* Annual precipitation reconstruction since AD 775 based on tree rings from the Qilian Mountains northwestern China. *Int J Climatlo* 2011; **31:** 371–381.
101. Liu Y, Lei Y and Sun B  *et al.* Annual precipitation in Liancheng China since 1777 AD derived from tree rings of Chinese pine(Pinus tabulaeformis Carr). *Int J Biometeorol* 2012; **57:** 927–934.
102. Liu Y, Wang CY and Song HM *et al.* Tree-ring-based annual precipitation reconstruction in Kalaqin Inner Mongolia for the last 238 years. *Chin Sci Bull* 2011; **56:** 2995–3002.
103. Gao J, Shi Z and Xu L *et al.* Precipitation variability in Hulunbuir northeastern China since 1829 AD reconstructed from tree-rings and its linkage with remote oceans. *J Arid Environ* 2013; **95:** 14–21.
104. Chen F, Yuan YJ and Wei WS *et al.* Tree-ring-based reconstruction of precipitation in the Changling Mountains China since AD1691. *Int J Biometeorol* 2012; **56:** 765–774.
105. Wang YJ, Gao SY and Ma YZ *et al.* Annual precipitation variation reconstructed by tree-ring width since AD 1899 in the west part of Hedong sandy area of Ningxia. *Arid Land Geogr* 2010; **33:** 377–384.
106. Gao S, Lu R and Qiang M *et al.* Reconstruction of precipitation in the last 140 years from tree ring at south margin of the Tengger Desert China. *Chin Sci Bull* 2005; **50:** 2487–2492.
107. Li JB, Chen FHand Cook ER *et al.* Drought reconstruction for North Central China from tree rings: the value of the Palmer drought severity index. *Int J Climatol* 2007;**27:** 903–909.
108. Tian QH, Gou XH and Zhang Y *et al.* Tree-ring based drought reconstruction ad 1855–2001 for the Qilian mountains northwestern China. *Tree-ring Res* 2007; **63:** 27–36.
109. Chen F, Yuan YJ and Wei WS  *et al.* Reconstruction of May–June Palmer drought severity index at south margin of Tengger Desert, China Since A.D.1691. *Scientia Geographica Sinica* 2011; **31:** 434–439.
110. Lu R, Gao S and Wang Y *et al.* Tree-ring based drought reconstruction at the northwestern margin of monsoon region of China since 1862. *Quatern Int* 2013; **283:** 93–97.
111. Chen F, Yuan YJ and Wei WS *et al.* Long-term drought severity variations recorded in tree rings in the northern Helan Mountains. *Adv Clim Change Res* 2010; **6:** 344–348.
112. Liang EY, Shao XM and Liu HY *et al.* Tree-ring based PDSI reconstruction since AD 1842 in the Ortindag Sand Land east Inner Mongolia. *Chin Sci Bull* 2007; **52:** 2715–2721.
113. Wang YJ, Lu RJ and Ma YZ *et al.* Annual variation in PDSI since 1897 AD in the Tengger Desert Inner Mongolia China as recorded by tree-ring data. *J Arid Environ* 2013; **98:** 20–26.
114. Deng Y, Gou XH and Gao LL *et al.* Aridity changes in the eastern Qilian Mountains since AD 1856reconstructed from tree-rings. *Quatern Int* 2013; **283:** 78–84.
115. Liu Y, An ZS and Linderholm HM *et al.* Annual temperatures during the last 2485 years in the mid-eastern Tibetan Plateau inferred from tree rings. *Sci China Ser D* 2009; **52:** 348–359.
116. Sun JY and Liu Y. Drought variations in the middle Qilian Mountains northeast Tibetan Plateau over the last 450 years as reconstructed from tree rings. *Dendrochronologia* 2013; **31:** 279–285.
117. Liu XH, Qin DH and Shao XM *et al.* Temperature variations recovered from tree-rings in the middle Qilian Mountain over the last millennium. *Sci China Ser D* 2005; **48:** 521–529.
118. Cai QFand Liu Y. Temperature variability since 1776 inferred from tree-rings of Pinus tabulaeformis in Mt. Helan. *Acta Geogr Sin* 2006; **61:** 929–936.
119. Zhu XD, Wang ZY and Lin LI *et al.* Summer temperature in northeast of Qaidam Basin retrieved from tree-ring chronology. *Sci Geograph Sin* 2007; **27**256-260.
120. Chen ZJ, Zhang X and He X *et al.* Extension of summer(June–August) temperature records (1715–2008) for northern Inner Mongolia China using tree rings. *Quatern Int* 2013; **283:** 21–29.
121. Kang XC, Zhang QH and Graumlich LJ *et al.* Reconstruction of a 1835 years past climate for Dulan Qinghai Province using tree-ring. *J Glaciol Geocryol* 2000; **22:** 65–72.
122. Zhu HF, Zheng YH and Shao XM *et al.* Millennial temperature reconstruction based on tree-ring widths of Qilian juniper from Wulan Qinghai Province China. *Chin Sci Bull* 2008; **53:** 3914–3920.
123. Liu Y, Xiang N and Song HM. Tree-ring temperature records in Arxan Inner Mongolia for the past 187 years. *J Earth Environ* 2012; **3:** 862–867.
124. Li JB, Gou XH and Cook ER *et al.* Tree-ring based drought reconstruction for the central Tien Shan area in northwest China. *Geophys Res Lett* 2006; **330:** 408–412.
125. Zhang TW, Yuan YJ and Liu Y *et al.* A tree-ring based temperature reconstruction for the Kaiduhe River watershed northwestern China since AD 1680: Linkages to the North Atlantic Oscillation. *Quatern Int* 2013; **311:** 71–80.
126. Chen F, Yuan YJ and Wei WS *et al.* Spring mean maximum temperature series and its variation properties in Hutubi River basin during the last 313 years. *J Desert Res* 2009; **29:** 162–167.
127. Chen F, Yuan YJ and Wei WS *et al.* Variation and prediction trend of precipitation series for the Tekes River basin during the last 236 years. *J Mountain Sci* 2010; **28:** 545–551.
128. Chen F, Yuan YJ and Wei WS *et al.* Reconstruction and analysis of precipitation in the Hutubi River basin on the northern slope of the Tianshan Mountains during the last 313 years. *Arid Zone Res* 2010; **26:** 130–135.
129. Chen F, Yuan YJ and Wei WS *et al.* Variations of long-term Palmer drought index in recent 354 years in Yili based on tree-ring record. *Plateau Meteorol* 2011; **30:** 355–362.
130. Cui Y, Yuan YJ and Jin HL *et al.* Reconstruction and analysis of 467–year spring precipitation series in the Urumqi River Head. *Arid Land Geogr* 2007; **30:** 496–500.
131. Hu YC, Yuan YJ and Wei WS *et al.* Tree-ring reconstruction of mean June–July temperature during 1613–2006 in East Altay Xinjiang of China. *J Desert Res* 2012;**32:** 1003–1009.
132. Lin J, Yuan YJ and Wei WS  *et al.* Utilizing tree-ring chronologies to reconstruct precipitation series at the north slope of western Tianshan Mountains. *J Desert Res*2013; **33:** 1527–1535.
133. Shang HM, Wei WS and Yuan YJ *et al.* Variations of temperature during last 350 years at Xinyuan recorded by tree-ring. *J Arid Land Resour Environ* 2011; **25:** 187–190.
134. Shang HM, Wei WS and Yuan YJ *et al.* The 150–year precipitation change recorded by tree ring in the central Tianshan Mountains. *Arid Zone Res* 2010; **27:** 443–449.
135. Shang HM, Wei WS and Yuan YJ *et al.* The mean June temperature history of 436a in Altay reconstructed from tree ring. *J Arid Land Resour Environ* 2010; **24:** 116–121.
136. Xu GB, Liu XH and Chen T *et al.* Temperature variations recorded in tree-ring width at timberline forest in Hami Badashi Xinjiang. *J Mountain Sci* 2009; **27:** 402–410.
137. Yu SL, Yuan YJ and Qin L *et al.* Reconstruction and analysis of the minimum temperature in summer for the Shawan in the Tianshan Mountains. *J Earth Environ* 2012; **3:** 868–873.
138. Yu SL, Yuan YJ and Jin HL *et al.* A 379–year July–August precipitation series reconstructed from tree-ring on the midwestern part of the northern slopes of Tianshan Mountains. *J Glaciol Geocryol* 2005; **65:** 1030–1030.
139. Yu SL, Yuan YJ and He Q *et al.* Reconstruction of temperature series from AD1468—2001 in the Jinghe Xinjiang. *J Glaciol Geocryol* 2007; **29:** 374–379.
140. Yuan YJ and Li J. Reconstruction and analysis of 450 years' winter temperature series in the Urumqi River source of Tianshan Mountains. *J Glaciol Geocryol* 1999; **21:** 64–70.
141. Yuan YJ, Shao XM and Li JF *et al.* Discussion of precipitation information in Xiagansate tree-ring chronology and 326 year precipitation reconstruction. *Acta Ecolog Sin* 2002; **22:** 2048–2053.
142. Yuan YJ, Wei YE and Dong GR. Reconstruction and discussion of 314 years precipitation in Yili prefecture western Tianshan Mountains. *J Glaciol Geocryol* 2000; **22:** 121–127.
143. Yuan YJ, Li JF and Hu RJ *et al.* Reconstruction of precipitation in the recent 350 years from tree-rings in the middle Tianshan Mountains. *J Glaciol Geocryol* 2001;**23:** 34–40.
144. Zhang L, Yuan YJ and Wei WS *et al.* Reconstruction and analysis of the 336a July and August precipitation series in Nilka county Xinjiang. *J Glaciol Geocryol* 2010; **32:** 914–920.
145. Zhang RB, Wei WS and Yuan YJ *et al.* A precipitation series of AD1396—2005 in Aksu River basin on the southern slopes of Tianshan Mountains: reconstruction and analysis. *J Glaciol Geocryol* 2009; **31:** 27–33.
146. Zhang RB, Yuan YJ and Wei WS *et al.* Monthly mean temperature from February to March in Aksu River basin on southern slope of Tianshan Mountain recorded by tree ring. *Plateau Meteorol* 2012; **31:** 804–809.
147. Zhang TW, Liu Y and Yuan YJ *et al.* Tree ring based mean maximum temperature reconstruction for the Gongnaisi region on the southern slope of the central Tianshan Mountains China since AD 1777. *Quatern Sci* 2011; **31:** 1011–1021.
148. Zhang TW, Yuan YJ and Yu SL *et al.* June to September precipitation series of 1481–2004 reconstructed from tree-ring in the western region of Altay prefecture Xinjiang. *J Glaciol Geocryol* 2008; **30:** 659–667.
149. Zhang TW, Wang LL and Yuan YJ *et al.* A 645–year precipitation reconstruction in Baluntai region on southern slope of mid–Tianshan Mountains based on tree-ring width. *Sci Geograph Sin* 2011; **31:** 251–256.
150. Zhang TW, Yuan YJ and Yu SL *et al.* Development of two tree-ring width chronologies of the samples collected from the Baluntai region. *Arid Zone Res* 2008; **25:** 288–294.
151. Cai QF and Liu Y. Two centuries temperature variations over subtropical southeast China inferred from Pinus taiwanensis Hayata tree-ring width. *Clim Dynam* 2016; **48:** 1–13.
152. Ren JL, Liu Y and Song HM *et al.* The historical reconstruction of the maximum temperature over the past 195 years Linxia region Gansu Province——Based on the data from Picea purpurea Mast. *Quatern Sci* 2014; **34:** 1270–1279.
153. Zhang RB, Yuan YJ and Wei WS *et al.* Dendroclimatic reconstruction of autumn–winter mean minimum temperature in the eastern Tibetan Plateau since 1600 AD. *Dendrochronologia* 2015; **33:** 1–7.
154. Liu Y, Zhang YH and Song HM *et al.* The tree-ring width based seasonal minimum temperature reconstruction at Shiren Mountains Henan China since 1850 AD and its record of 20th century warming. *J Earth Environ* 2015; **6:** 393–406.
155. Cai QF, Liu Y and Wang YC *et al.* Recent warming evidence inferred from a tree-ring-based winter–half year minimum temperature reconstruction in northwestern Yichang South Central China and its relation to the large-scale circulation anomalies. *Int J Biometeorol* 2016; **60:** 1885–1896.
156. Lin J, Yuan YJ and Wei WS *et al.* Mean minimum temperature change recorded in the tree-ring in the middle and east of Boertala Mongolia Autonomous Prefecture. *J Arid Land Resour Environ* 2014; **28:** 166–172.
157. Lei Y, Liu Y and Sun B *et al.* Interannual variability of average minimum temperatures derived from tree rings in the mid–Qinling Mountains China for the past 138 years. *Int J Biometeorol* 2016; **60:** 1519–1529.
158. Zhang Y, Shao XM and Yin ZY *et al.* Millennial minimum temperature variations in the Qilian Mountains China: evidence from tree rings. *Clim Past* 2014; **10:** 1763–1778.
159. Shi CM, Masson-Delmotte V and Daux V *et al.* Unprecedented recent warming rate and temperature variability over the east Tibetan Plateau inferred from Alpine tree line dendrochronology. *Clim Dynam*2015; **45:** 1367–1380.
160. Lv SN and Wang XC. Growth–climate response and winter precipitation reconstruction of Pinus sylvestris var mongolicain A'li River of Greater Khingan Range. *J Northeast Normal Univ* 2014; **46:** 110–116.
161. Shi JF, Lu HY and Li J *et al.* Tree-ring based February–April precipitation reconstruction for the lower reaches of the Yangtze River southeastern China. *Global Planet Change* 2015; **131:** 82–88.
162. Chen F, Wang H and Chen FH  *et al.* Tree-ring reconstruction of July–May precipitation(AD 1816–2010) in the northwestern marginal zone of the East Asian summer monsoon reveals the monsoon-related climate signals. *Int J Climatol* 2015; **35:** 2109–2121.
163. Liu JJ. Precipitation variations during the last 526 years inferred from tree-ring widths on the Southern Tibetan Plateau. *J Lanzhou Univ(Nat Sci)*2014; **50:** 293–298.
164. Jiang SX, Yuan YJ and Chen F *et al.* A 291 year precipitation reconstruction in the Upper Irtysh river basin based on tree-ring width. *Acta Ecolog Sin*2016; **36:** 2866–2875.
165. Chen F, Yuan JY and Wei WS *et al.* Precipitation reconstruction for the southern Altay Mountains(China) from tree rings of Siberian spruce reveals recent wetting trend. *Dendrochronologia* 2014; **32:** 266–272.
166. Zhang TW, Yuan YJ and Wei WS *et al.* A tree-ring based precipitation reconstruction for the Mohe region in the northern Greater Higgnan Mountains China since AD 1724.*Quatern Res* 2014; **82:** 14–21.
167. Fang KY, Wilmking M and Davi N *et al.* An ensemble weighting approach for dendroclimatology: drought reconstructions for the northeastern Tibetan Plateau. *Plos One*2014; **9:** e86689.
168. Zhang YH, Liu Y and Song HM *et al.* Tree-ring-based seasonal precipitation reconstruction in Mt Shennong for the last 162 years. *J Earth Environ* 2013; **4:** 1450–1460.
169. Peng JF, Liu YZ and Wang T. A tree-ring record of 1920's—1940's droughts and mechanism analyses in Henan Province. *Acta Ecolog Sini* 2014; **34:** 3509–3518.
170. Chen F, Yuan YJ and Zhang RB *et al.* A tree-ring based drought reconstruction(AD 1760–2010) for the Loess Plateau and its possible driving mechanisms. *Global Planet Change*2014; **122:** 82–88.
171. Liu Y, Zhang XJ and Song HM *et al.* Tree-ring-width-based PDSI reconstruction for central Inner Mongolia China over the past 333 years. *Clim Dynam*. 2016; **48:** 867–879
172. Cai QF, Liu Y and Lei Y  *et al.* Reconstruction of the March-August PDSI since 1703 AD based on tree rings of Chinese pine (Pinus tabulaeformis Carr) in the Lingkong Mountain southeast Chinese Loess Plateau. *Clim Past* 2014; **10:** 509–521.
173. Gou XH, Gao LL and Deng Y *et al.* An 850–year tree-ring-based reconstruction of drought history in the western Qilian Mountains of northwestern China. *Int J Climatol* 2014; **35:** 3308–3319.
174. Cai QF, Liu Y and Liu H *et al.* Reconstruction of drought variability in North China and its association with sea surface temperature in the joining area of Asia and Indian–Pacific Ocean. *Palaeogeogr Palaeocl Palaeoec* 2015; **417:** 554–560.
175. Li Q, Liu Y and Song HM *et al.* Divergence of tree-ring-based drought reconstruction between the individual sampling site and the Monsoon Asia Drought Atlas An example from Guancen Mountain. *Sci Bull* 2015; **19:** 1688–1697.
176. Chen F, Yu SL and Yuan YJ *et al.* A tree-ring width based drought reconstruction for southeastern China: links to Pacific Ocean climate variability. *Boreas* 2016; **45:** 335–346.
177. Zhang TW Yuan YJ and Liu Y *et al.* A tree-ring based precipitation reconstruction for the Baluntai region on the southern slope of the central Tien Shan Mountains, China, since A.D. 1464. Quaternary International 2013；283:55–62.
178. Li ZS, Liu GH and Wu X *et al.* Tree-ring-based temperature reconstruction for the Wolong Natural Reserve western Sichuan Plateau of China. *Int J Climatol* 2014; **35:** 3296–3307.
179. Zheng YH, Shao XM and Lu F *et al.* February–May temperature reconstruction based on tree-ring widths of Abies fargesii from the Shennongjia area in central China. *Int J Biometeorol* 2016; **60:** 1175–1184.
180. Chen F, Zhang RB and Wang HQ *et al.* Recent climate warming of central China reflected by temperature-sensitive tree growth in the eastern Qinling Mountains and its linkages to the Pacific and Atlantic Oceans. *J Mountain Sci* 2015; **12:** 396–403.
181. Chen F, Yuan JY and Wei WS *et al.* Tree-ring based temperature reconstruction for the west Qinling Mountains (China): linkages to the High Asia solar activity and Pacific–Atlantic Ocean. *Geochronometria* 2014; **41:** 234–244.
182. Song HM, Liu Y and Li Q *et al.* Tree-ring based May–July temperature reconstruction since AD 1630on the western Loess Plateau China. *Plos One* 2014; **9:** e93504.
183. Shi JF, Li LL and Han ZY *et al.* Tree-ring width based June–September temperature reconstruction and its teleconnection with PDO and ENSO in Mount Daowu Hunan Province. *Quatern Sci* 2015; **35:** 1155–1164.
184. Li JJ, Shao XM and Li YY *et al.* Annual temperature recorded in tree-ring from Songpan region. *Chin Sci Bull* 2014; **59:** 1446–1458.
185. Zhang QB, Evans MN and Lv LX. Moisture dipole over the Tibetan Plateau during the past five and a half centuries. *Nat Commun 2015;* **6:**8062.
